# Disentangling the causal relationship between rabbit growth and cecal microbiota through structural equation models

**DOI:** 10.1186/s12711-022-00770-2

**Published:** 2022-12-19

**Authors:** Mónica Mora, María Velasco-Galilea, Juan Pablo Sánchez, Yuliaxis Ramayo-Caldas, Miriam Piles

**Affiliations:** 1 Institute of Agrifood Research and Technology (IRTA)-Animal Breeding and Genetics, Caldes de Montbui, Barcelona Spain; 2 Centre for Research in Agricultural Genomics (CRAG), CSIC-IRTA-UAB-UB, Cerdanyola del Vallès, Barcelona Spain

## Abstract

**Background:**

The effect of the cecal microbiome on growth of rabbits that were fed under different regimes has been studied previously. However, the term “effect” carries a causal meaning that can be confounded because of potential genetic associations between the microbiome and production traits. Structural equation models (SEM) can help disentangle such a complex interplay by decomposing the effect on a production trait into direct host genetics effects and indirect host genetic effects that are exerted through microbiota effects. These indirect effects can be estimated via structural coefficients that measure the effect of the microbiota on growth while the effects of the host genetics are kept constant. In this study, we applied the SEM approach to infer causal relationships between the cecal microbiota and growth of rabbits fed under ad libitum (ADG_AL_) or restricted feeding (ADG_R_).

**Results:**

We identified structural coefficients that are statistically different from 0 for 138 of the 946 operational taxonomic units (OTU) analyzed. However, only 15 and 38 of these 138 OTU had an effect greater than 0.2 phenotypic standard deviations (SD) on ADG_AL_ and ADG_R_, respectively. Many of these OTU had a negative effect on both traits. The largest effects on ADG_R_ were exerted by an OTU that is taxonomically assigned to the *Desulfovibrio* genus (− 1.929 g/d, CSS-normalized OTU units) and by an OTU that belongs to the *Ruminococcaceae* family (1.859 g/d, CSS-normalized OTU units). For ADG_AL_, the largest effect was from OTU that belong to the *S24-7* family (− 1.907 g/d, CSS-normalized OTU units). In general, OTU that had a substantial effect had low to moderate estimates of heritability.

**Conclusions:**

Disentangling how direct and indirect effects act on production traits is relevant to fully describe the processes of mediation but also to understand how these traits change before considering the application of an external intervention aimed at changing a given microbial composition by blocking/promoting the presence of a particular microorganism.

**Supplementary Information:**

The online version contains supplementary material available at 10.1186/s12711-022-00770-2.

## Background

Gut microbiota influence the metabolism of the host and, therefore, its growth and feed efficiency (FE) [1–3], which are key components of profitability, productivity, and sustainability in the meat production industry. Feed efficiency can be measured as average daily gain under a restricted feeding regime (ADG_R_) since variation in growth rate under restricted feeding is directly related to variation in FE because feed intake (FI) is constant [1, 4]. On the other hand, average daily gain under ad libitum feeding (ADG_AL_) reflects an animal’s ability to grow. Both, ADG_AL_ and ADG_R_ are heritable traits, which implies that host genetics plays an important role in regulating growth and feed utilization [5]. Thus, these traits have been successfully used as selection criteria in experiments carried out in pigs and rabbits [4, 6]. In addition, a genome-wide association study (GWAS) on rabbits conducted by Sánchez et al. [7] identified different quantitative trait loci (QTL) for both these traits, supporting the idea that the genetic control of the growth of animals fed under a restricted regime differs from that of animals fed ad libitum. This result is also supported by the estimate of the genetic correlation of 0.59 between these traits that was reported by Piles and Sánchez [5]. In line with results of previous research in human [8] and livestock [9–11] populations, a recent study conducted by Velasco-Galilea et al. [12] in the same rabbit population as used in [7] suggested that, in rabbit, cecal microbial composition and diversity are heritable traits, and identified several host genomic regions that affect cecal microbial composition. However, since most of these studies are based on associations or correlations, causal relationships between the microbiome and the traits cannot be implied. Thus, which fractions of the genetic effect on a phenotypic trait (such as ADG_AL_ and ADG_R_) are exerted directly versus indirectly, e.g. by promoting a specific microbial profile in the gut that has a favourable or unfavourable effect on the trait, remains unknown. As Gardiner et al. [3] pointed out in their review: “some specific examples are outlined where an increased abundance of certain microbiota members may be a result of improved productivity traits rather than the cause of them”. In addition, when analysing the impact of the microbiome on a host phenotype, this effect can be confounded with host genetics effects if the genetic association between the microbiome and the phenotype is not considered in the model. How important these indirect host genetics effects that are exerted through the gut microbiota are relative to the direct genetic effects on growth and FE has not been evaluated in rabbits. Direct and indirect effects could be opposite in sign, leading to a null effect of the genotype on the trait if they are of similar magnitude. This could be the case of a microorganism that has a negative effect on a phenotypic trait and for which its relative abundance is associated with a host genetic marker that is linked to a gene that positively affects the same trait through some metabolic process. If both effects are of similar magnitude, the effect of that marker would not be captured by a standard GWAS for the trait. Structural equation models (SEM) [13] can disentangle the complex interplay that exists between the host genome and its microbiome. SEM can also help to predict the effect that an external intervention would have on the system, such as one directed at inhibiting or blocking the effect of certain microorganisms. Multiple-trait animal models (MTAM) capture correlations or associations among traits but do not inform about their causal relationship. In contrast, SEM allow the decomposition of the total effect (genetic and environmental) on a trait into direct and indirect contributions. Thus, SEM can provide better insights in the relationships and the biological mechanisms among traits than MTAM.

The objectives of this research were: (1) to identify the operational taxonomic units (OTU) with abundances in the fecal microbiome that impact rabbit growth (ADG_AL_ and ADG_R_), (2) to quantify the relative importance of direct and microbial-mediated host genetic effects on ADG_AL_ and ADG_R_, (3) to estimate the heritability of the abundance of the most important OTU, and (4) to assess the importance of direct genetic relative to total genetic covariances of ADG_AL_ and ADG_R_ with abundance of OTU.

## Methods

### Animals and phenotypic data

The 412 animals included in the present study belong to a line of rabbits that has been selected for post-weaning growth since 1983 and that is commonly used as a terminal sire line in the three-way crossbreeding scheme for rabbit meat production [14]. They were randomly selected from five batches of a larger experiment [5]. Most were produced in four batches in a semi-open-air facility of the Institute of Agrifood Research and Technology (IRTA, Barcelona Spain) during the first semester of 2014, and the remaining were produced in a single batch in another facility of IRTA under controlled environmental conditions in the spring of 2016. The animals bred in the first facility were housed in groups of eight kits from weaning (32 days of age) until the end of the fattening period (66 days of age). The kits raised in the second facility were housed in groups of six kits and their growing period was slightly shorter (from 32 to 60 days of age). A maximum of two kits from the same litter were assigned to the same cage to avoid confounding between cage and maternal effects. Apart from the afore-mentioned differences, all animals were raised under the same management conditions and were fed with a standard pelleted diet supplemented with antibiotics, except for 23 kits raised in 2016 that received the same food but free of antibiotics. Water was provided ad libitum and feed was supplied once per day in a feeder with three places during the 4- to 5-week fattening period. At weaning, the animals were randomly assigned to the ad libitum (AL) or restricted (R) feeding regime. The amount of feed supplied to the animals under R during each week for each batch was computed as 0.75 times the average FI of kits under AL from the same batch during the previous week, plus 10% to account for the increase in feed intake as the animals grow [15]. To generate homogeneous groups, kits under each feeding regime were categorized into two groups according to their individual weaning weight (WW) being greater or smaller than 700 g.

Individual body weight (BW) was recorded weekly for all animals. Individual ADG was computed as the slope of the within-animal regression of BW on age. This trait was computed for individuals under each feeding regime, thus obtaining ADG under AL (ADG_AL_) and R (ADG_R_). The number of animals and descriptive statistics for both traits are in Table 1.

**Table 1:** Descriptive statistics of average daily gain under ad libitum (ADG_AL_) and restricted (ADG_R_) feeding

Trait	N	Mean	SD	IQR
ADG_AL_ (g/day)	218	55.09	5.91	7.57
ADG_R_ (g/day)	194	38.84	5.27	6.89

*N* number of animals, *SD* standard deviation, *IQR* interquartile range

### Cecal sampling, microbial DNA extraction, and bioinformatic processing

For each animal, a cecal sample was collected in a sterile tube immediately after slaughter, kept cold in the laboratory (4 °C) and, stored at – 80 °C until DNA extraction. Full details of the DNA extraction, amplification, library preparation and sequencing are given in previous reports [16, 17]. Briefly, DNA integrity/purity was checked according to the protocol of Desjardins and Conklin [18], and then DNA was amplified following Parada et al. [19].

Raw reads were processed with the QIIME software version 1.9.0 (https://github.com/biocore/qiime/releases/tag/1.9.0) [20], as described in [16]. Contigs with a quality score smaller than Q19 were removed. The UCHIME algorithm [21] was used to detect and remove chimeric sequences. Filtered sequences were clustered into OTU and those that were detected in less than 5% of the samples and with a count sum less than 0.01% were discarded. The remaining 946 OTU were normalized using the cumulative sum scaling (CSS) method [22]. Taxonomic assignment of each OTU was obtained by mapping the sequences against the Greengenes reference database. Raw sequence data were deposited in the sequence read archive of NCBI under BioProject accession number PRJNA524130.

### Host DNA extraction and single nucleotide polymorhism (SNP) genotyping

Using the MN Nucleospin Tissue kit (Macherey-Nagel, Germany), host DNA was extracted from liver samples collected at slaughter. DNA integrity and purity were measured following the protocol of Desjardins and Conklin [18]. The Affymetrix Axiom OrcunSNP array (199,692 SNPs) was used to genotype 412 rabbits. Quality control of the SNPs was performed with the PLINK software (version 1.9) [23] according to four criteria: (i) individual call rate > 0.90; (ii) SNP call rate > 0.95; (iii) SNP minor allele frequency (MAF) > 0.05; (iv) and only autosomal SNPs with known positions in the OryCun2.0 assembly [24] were used. The final dataset contained genotypes for 114,604 SNPs on 412 rabbits.

### Statistical analyses

**Fig. 1:**
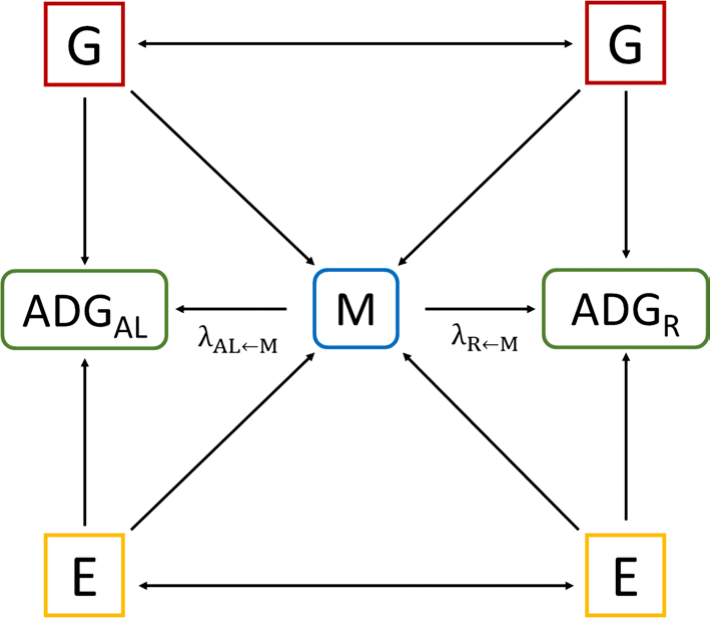
Graphical representation of the structural equations model. ADG_AL_: average daily gain of growing rabbits fed ad libitum*,* ADG_R_: average daily gain of growing rabbits under restricted feeding, M: microbiome, G: host genetics, E: environmental factors, ${\uplambda }_{\mathrm{AL}\leftarrow \mathrm{M}}$: structural coefficient of the effect of M on ADG_AL_ and ${\uplambda }_{\mathrm{R}\leftarrow \mathrm{M}}$: structural coefficient of the effect of M on ADG_R_

With the main objective of estimating the effect of the microbiota on growth, while taking into account that these two traits are genetically correlated, the SEM represented in Fig. 1 was implemented to assess the causal relationship of each OTU (M) with ADG_AL_ and ADG_R_. In this model, the host genotype directly affects the phenotypes (G → ADG_AL_ and G → ADG_R_) but also the gut microbiome (G → M), while, the microbiota also affects the phenotypes (G → M → ADG_AL_ and G → M → ADG_R_). In Fig. 1, ${\uplambda }_{\mathrm{AL}\leftarrow \mathrm{M}}$ and ${\uplambda }_{\mathrm{R}\leftarrow \mathrm{M}}$ are the structural coefficients and indicate the effect of M on ADG_AL_ and ADG_R_, respectively, while keeping G constant. Environmental factors (E) affect M and the phenotypes.

For each OTU, the SEM was implemented using a Bayesian analysis of three phenotypes: ADG_AL_, ADG_R_, and OTU (referred as M). The analysis was repeated for each OTU, i.e. 946 times. The SEM for ADG_AL_, ADG_R_ and the m^th^ OTU (m = 1, 2, …, 946) for the $\mathrm{n}$ animals can be written as:

1 $$\mathbf{y}=\left({\varvec{\Lambda}}\otimes{\mathbf{I}}_{\mathrm{n}}\right)\mathbf{y}+\mathbf{X}\mathbf{b}+{\mathbf{Z}}_{\mathbf{l}}\mathbf{l}+{\mathbf{Z}}_{\mathbf{c}}\mathbf{c}+{\mathbf{Z}}_{\mathbf{u}}\mathbf{u}+\mathbf{e},$$

where $\mathbf{y}$ is the vector of phenotypic records containing observations for ADG_AL_, ADG_R_, and M (with ADG_R_ missing for animals on AL and ADG_AL_ missing for animals on R); ${\varvec{\Lambda}}$ is the matrix of structural coefficients, defined as:

2 $${\varvec{\Lambda}}= \left[\begin{array}{ccc}0& 0& {\uplambda }_{\mathrm{AL}\leftarrow \mathrm{M}}\\ 0& 0& {\uplambda }_{\mathrm{R}\leftarrow \mathrm{M}}\\ 0& 0& 0\end{array}\right],$$

where ${\uplambda }_{\mathrm{AL}\leftarrow \mathrm{M}}$ and ${\uplambda }_{\mathrm{R}\leftarrow \mathrm{M}}$ are the effect of M on ADG_AL_ and ADG_R_, respectively; $\mathbf{b}$ is the vector of systematic effects, with incidence matrix $\mathbf{X}$, which included the effects of the combination of batch and farm (5 levels) and of bodyweight at weaning (2 levels: large and small); $\mathbf{l}$ and $\mathbf{c}$ are the vectors of permanent environmental effects of litter (195 levels) and cage (189 levels), respectively; $\mathbf{u}$ is the vector of genotypic effects for the 412 animals, and $\mathbf{e}$ is the vector of model residuals. The vectors $\mathbf{l}$, $\mathbf{c}$, $\mathbf{u},$ and $\mathbf{e}$ are assumed to follow a joint distribution:

3 $$\left[\begin{array}{c}\mathbf{l}\\ \mathbf{c}\\ \mathbf{u}\\ \mathbf{e}\end{array}\right] \sim N \left\{\left[\begin{array}{c}\bf{0}\\ \bf{0}\\ \bf{0}\\ \bf{0}\end{array}\right],\left[\begin{array}{cccc}{\mathbf{L}}_{\bf{0}}{\otimes\mathbf{I}}_{\mathrm{n}}& \bf{0}& \bf{0}& \bf{0}\\ \bf{0}& {\mathbf{C}}_{\bf{0}}\otimes{\mathbf{I}}_{\mathrm{n}}& \bf{0}& \bf{0}\\ \bf{0}& \bf{0}& {\mathbf{G}}_{\bf{0}}\otimes\mathbf{G}& \bf{0}\\ \bf{0}& \bf{0}& \bf{0}& {\mathbf{R}}_{\bf{0}}\otimes{\mathbf{I}}_{\mathrm{n}}\end{array}\right]\right\},$$

where $\mathbf{G}$ is the genomic relationship matrix and ${\mathbf{L}}_{0}$, ${\mathbf{C}}_{0}$, ${\mathbf{G}}_{0},$ and ${\mathbf{R}}_{0}$ are symmetric covariance matrices for litter, cage, genomic and residual effects, respectively, with elements equal to (only the upper triangular matrix is shown):

$${\mathbf{L}}_{0}=\left[\begin{array}{ccc}{\upsigma }_{\mathrm{l},\mathrm{AL}}^{2}& {\upsigma }_{\mathrm{l},\mathrm{AL},\mathrm{R}}& {\upsigma }_{\mathrm{l},\mathrm{AL},\mathrm{M}}\\ & {\upsigma }_{\mathrm{l},\mathrm{R}}^{2}& {\upsigma }_{\mathrm{l},\mathrm{R},\mathrm{M}}\\ & & {\upsigma }_{\mathrm{l},\mathrm{M}}^{2}\end{array}\right],$$

$${\mathbf{C}}_{0}=\left[\begin{array}{ccc}{\upsigma }_{\mathrm{c},\mathrm{AL}}^{2}& {\upsigma }_{\mathrm{c},\mathrm{AL},\mathrm{R}}& {\upsigma }_{\mathrm{c},\mathrm{AL},\mathrm{M}}\\ & {\upsigma }_{\mathrm{c},\mathrm{R}}^{2}& {\upsigma }_{\mathrm{c},\mathrm{R},\mathrm{M}}\\ & & {\upsigma }_{\mathrm{c},\mathrm{M}}^{2}\end{array}\right],$$

$${\mathbf{G}}_{0}=\left[\begin{array}{ccc}{\upsigma }_{\mathrm{u},\mathrm{AL}}^{2}& {\upsigma }_{\mathrm{u},\mathrm{AL},\mathrm{R}}& {\upsigma }_{\mathrm{u},\mathrm{AL},\mathrm{M}}\\ & {\upsigma }_{\mathrm{u},\mathrm{R}}^{2}& {\upsigma }_{\mathrm{u},\mathrm{R},\mathrm{M}}\\ & & {\upsigma }_{\mathrm{u},\mathrm{M}}^{2}\end{array}\right],$$

and $${\mathbf{R}}_{0}=\left[\begin{array}{ccc}{\upsigma }_{\mathrm{e},\mathrm{AL}}^{2}& 0& 0\\ & {\upsigma }_{\mathrm{e},\mathrm{R}}^{2}& 0\\ & & {\upsigma }_{\mathrm{e},\mathrm{M}}^{2}\end{array}\right]$$. Note that, the parameters of the SEM are identifiable for any acyclic structure among traits, because ${\mathbf{R}}_{0}$ is diagonal [25].

Solving Eq. (1), the SEM becomes:

4 $$\left({\mathbf{I}}_{\mathbf{3n}}-{\varvec{\Lambda}}\otimes{\mathbf{I}}_{\mathrm{n}}\right)\mathbf{y}=\mathbf{X}\mathbf{b}+{\mathbf{Z}}_{\mathrm{l}}\mathbf{l}+{\mathbf{Z}}_{\mathrm{c}}\mathbf{c}+{\mathbf{Z}}_{\mathrm{u}}\mathbf{u}+\mathbf{e},$$

where ${\mathbf{I}}_{\mathbf{3n}}$ is a (3n × 3n) identity matrix.

Thus, the reduced model is:

5 $$\begin{aligned}\mathbf{y}&={\left({\mathbf{I}}_{\mathbf{3n}}-{\varvec{\Lambda}}\otimes{\mathbf{I}}_{\mathrm{n}}\right)}^{-1}\mathbf{X}\mathbf{b} +{\left({\mathbf{I}}_{\mathbf{3n}}-{\varvec{\Lambda}}\otimes{\mathbf{I}}_{\mathrm{n}}\right)}^{-1}{\mathbf{Z}}_{\mathrm{l}}\mathbf{l} \\ &\quad+{{\left({\mathbf{I}}_{\mathbf{3n}}-{\varvec{\Lambda}}\otimes{\mathbf{I}}_{\mathrm{n}}\right)}^{-1}\mathbf{Z}}_{\mathrm{c}}\mathbf{c}+{\left({\mathbf{I}}_{\mathbf{3n}}-{\varvec{\Lambda}}\otimes{\mathbf{I}}_{\mathrm{n}}\right)}^{-1}{\mathbf{Z}}_{\mathrm{u}}\mathbf{u}\\ &\quad+{\left({\mathbf{I}}_{\mathbf{3n}}-{\varvec{\Lambda}}\otimes{\mathbf{I}}_{\mathrm{n}}\right)}^{-1}\mathbf{e}\\ &= {\mathbf{X}\mathbf{b}}^{*}+{\mathbf{Z}}_{\mathrm{l}}{\mathbf{l}}^{\mathbf{*}}+{{\mathbf{Z}}_{\mathrm{c}}\mathbf{c}}^{\mathbf{*}}+{{\mathbf{Z}}_{\mathrm{u}}\mathbf{u}}^{\mathbf{*}}+{\mathbf{e}}^{\mathbf{*}},\end{aligned}$$

which corresponds to a MTAM with:

6 $$\left[\begin{array}{c}{\mathbf{l}}^{\mathbf{*}}\\ {\mathbf{c}}^{\mathbf{*}}\\ {\mathbf{u}}^{\mathbf{*}}\\ {\mathbf{e}}^{\mathbf{*}}\end{array}\right] \sim \mathrm{N }\left\{\left[\begin{array}{c}\bf{0}\\ \bf{0}\\ \bf{0}\\ \bf{0}\end{array}\right],\left[\begin{array}{cccc}{\mathbf{L}}_{\bf{0}}^{\mathbf{*}}\otimes {\mathbf{I}}_{\mathrm{n}}& \bf{0}& \bf{0}& \bf{0}\\ \bf{0} &{\mathbf{C}}_{\bf{0}}^{\mathbf{*}}\otimes {\mathbf{I}}_{\mathrm{n}}& \bf{0}& \bf{0}\\ \bf{0}& \bf{0}& {\mathbf{G}}_{\bf{0}}^{\mathbf{*}}\otimes \mathbf{G}& \bf{0}\\ \bf{0}& \bf{0}& \bf{0}& {\mathbf{R}}_{\bf{0}}^{\mathbf{*}}\otimes {\mathbf{I}}_{\mathrm{n}}\end{array}\right]\right\},$$

and

7 $${\mathbf{L}}_{0}^{*}={\left({\mathbf{I}}_{\mathbf{3}}-{\varvec{\Lambda}}\right)}^{-1}{{{\mathbf{L}}_{0}\left({\mathbf{I}}_{\mathbf{3}}-{\varvec{\Lambda}}\right)}^{-1}}^{^{\prime}},$$

8 $${\mathbf{C}}_{0}^{*}={\left({\mathbf{I}}_{\mathbf{3}}-{\varvec{\Lambda}}\right)}^{-1}{{{\mathbf{C}}_{0}\left({\mathbf{I}}_{\mathbf{3}}-{\varvec{\Lambda}}\right)}^{-1}}^{^{\prime}},$$

9 $${\mathbf{G}}_{0}^{*}={\left({\mathbf{I}}_{\mathbf{3}}-{\varvec{\Lambda}}\right)}^{-1}{{{\mathbf{G}}_{0}\left({\mathbf{I}}_{\mathbf{3}}-{\varvec{\Lambda}}\right)}^{-1}}^{^{\prime}},$$

and

10 $${\mathbf{R}}_{0}^{*}={\left({\mathbf{I}}_{\mathbf{3}}-{\varvec{\Lambda}}\right)}^{-1}{{{\mathbf{R}}_{0}\left({\mathbf{I}}_{\mathbf{3}}-{\varvec{\Lambda}}\right)}^{-1}}^{^{\prime}}.$$

In the SEM, ${\mathbf{u}}_{\mathrm{AL}}$, ${\mathbf{u}}_{\mathrm{R},}$ and ${\mathbf{u}}_{\mathrm{M}}$ (component vectors of u) represent the genomic effects that directly affect ADG_AL_, ADG_R_, and M, respectively. They can also be described as the effect of the genome on a trait, while holding the value for the remaining traits constant, i.e. the direct effects of the genome on a trait free from genetic effects that are mediated through other phenotypic traits that have a causal influence on the target trait [26]. In turn, ${\uplambda }_{\mathrm{AL}\leftarrow \mathrm{M}}{\mathbf{u}}_{\mathrm{M}}$ and ${\uplambda }_{\mathrm{R}\leftarrow \mathrm{M}}{\mathbf{u}}_{\mathrm{M}}$ represent the indirect genomic effects that are mediated by M on ADG_AL_ and ADG_R_, respectively, such that ${\mathbf{u}}_{\mathrm{AL}}^{*}={\uplambda }_{\mathrm{AL}\leftarrow \mathrm{M}}{\mathbf{u}}_{\mathrm{M}}+{\mathbf{u}}_{\mathrm{AL}}$ and ${\mathbf{u}}_{\mathrm{R}}^{*}={\uplambda }_{\mathrm{R}\leftarrow \mathrm{M}}{\mathbf{u}}_{\mathrm{M}}+{\mathbf{u}}_{\mathrm{R}}$ are the overall genetic effects exerted by the genome of an individual on ADG_AL_ and ADG_R_, respectively, through all the paths. Thus, the indirect effects on ADG_AL_ and ADG_R_ correspond to the direct effects of genes exerted over M, which, in turn, influence ADG_AL_ and ADG_R_.

Components of ${\mathbf{G}}_{0}^{*}$ can be written as:

11 $${\upsigma }_{{\mathrm{u}}^{*},\mathrm{AL}}^{2}={\upsigma }_{\mathrm{u},\mathrm{AL}}^{2}+2\times {\uplambda }_{\mathrm{AL}\leftarrow \mathrm{M}}\times {\upsigma }_{\mathrm{u},\mathrm{M},\mathrm{AL}}+{\uplambda }_{\mathrm{AL}\leftarrow \mathrm{M}}^{2}\times {\upsigma }_{\mathrm{u},\mathrm{M}}^{2},$$

12 $${\upsigma }_{{\mathrm{u}}^{*},\mathrm{R}}^{2}={\upsigma }_{\mathrm{u},\mathrm{R}}^{2}+{2\times\uplambda }_{\mathrm{R}\leftarrow \mathrm{M}}\times {\upsigma }_{\mathrm{u},\mathrm{M},\mathrm{R}}+{\uplambda }_{\mathrm{R}\leftarrow \mathrm{M}}^{2}\times {\upsigma }_{\mathrm{u},\mathrm{M}}^{2},$$

13 $${\upsigma }_{{\mathrm{u}}^{*},\mathrm{M}}^{2}={\upsigma }_{\mathrm{u},\mathrm{M}}^{2},$$

where ${\upsigma }_{{\mathrm{u}}^{*},\mathrm{AL}}^{2}$, ${\upsigma }_{{\mathrm{u}}^{*},\mathrm{R}}^{2},$ and ${\upsigma }_{{\mathrm{u}}^{*},\mathrm{M}}^{2}$ are the total genetic variance of ADG_AL_, ADG_R_, and M, respectively; ${\upsigma }_{\mathrm{u},\mathrm{AL}}^{2}$, ${\upsigma }_{\mathrm{u},\mathrm{R}}^{2},$ and ${\upsigma }_{\mathrm{u},\mathrm{M}}^{2}$ are the genetic variance of direct effects on ADG_AL_, ADG_R_, and M, respectively; and ${\upsigma }_{\mathrm{u},\mathrm{AL},\mathrm{M}}$ and ${\upsigma }_{\mathrm{u},\mathrm{R},\mathrm{M}}$ are the covariances between direct effects, which represent the effects of genes that directly affect both traits (i.e. pleitropic ADG_AL_ and ADG_R_ effects) or of genes that affect one of the two traits and are in linkage disequilibrium with each other, respectively. Structural coefficients ${\uplambda }_{\mathrm{AL}\leftarrow \mathrm{M}}$ and ${\uplambda }_{\mathrm{R}\leftarrow \mathrm{M}}$ were as defined above. And

14 $${\upsigma }_{{\mathrm{u}}^{*},\mathrm{AL},\mathrm{R}}={\upsigma }_{\mathrm{u},\mathrm{AL},\mathrm{R}}+{\uplambda }_{\mathrm{AL}\leftarrow \mathrm{M}}\times {\upsigma }_{\mathrm{u},\mathrm{M},\mathrm{R}}+{\uplambda }_{\mathrm{R}\leftarrow \mathrm{M}}\times {\upsigma }_{\mathrm{u},\mathrm{M},\mathrm{AL}}+{{\uplambda }_{\mathrm{R}\leftarrow \mathrm{M}}\times\uplambda }_{\mathrm{AL}\leftarrow \mathrm{M}}\times {\upsigma }_{\mathrm{u},\mathrm{M}}^{2},$$

15 $${\upsigma }_{{\mathrm{u}}^{*},\mathrm{AL},\mathrm{M}}={\upsigma }_{\mathrm{u},\mathrm{AL},\mathrm{M}}+{\uplambda }_{\mathrm{AL}\leftarrow \mathrm{M}}\times {\upsigma }_{\mathrm{u},\mathrm{M}}^{2},$$

and

16 $${\upsigma }_{{\mathrm{u}}^{*},\mathrm{R},\mathrm{M}}={\upsigma }_{\mathrm{u},\mathrm{R},\mathrm{M}}+{\uplambda }_{\mathrm{R}\leftarrow \mathrm{M}}\times {\upsigma }_{\mathrm{u},\mathrm{M}}^{2},$$

are the total covariance between ADG_AL_ and ADG_R_, between ADG_AL_ and M, and between ADG_R_ and M, respectively. Note that if ${\uplambda }_{\mathrm{AL}\leftarrow \mathrm{M}}\times {\upsigma }_{\mathrm{u},\mathrm{M}}^{2}$ and ${\uplambda }_{\mathrm{R}\leftarrow \mathrm{M}}\times {\upsigma }_{\mathrm{u},\mathrm{M}}^{2}$ have opposite signs but equal magnitude to ${\upsigma }_{\mathrm{u},\mathrm{AL},\mathrm{M}}$ and ${\upsigma }_{\mathrm{u},\mathrm{R},\mathrm{M}}$, respectively, the total genetic covariances between the ADG traits and M could be null. However, ${\upsigma }_{\mathrm{u},\mathrm{AL},\mathrm{R}}+{\uplambda }_{\mathrm{AL}\leftarrow \mathrm{M}}\times {\upsigma }_{\mathrm{u},\mathrm{M},\mathrm{R}}+{\uplambda }_{\mathrm{R}\leftarrow \mathrm{M}}\times {\upsigma }_{\mathrm{u},\mathrm{M},\mathrm{AL}}+{{\uplambda }_{\mathrm{R}\leftarrow \mathrm{M}}\times\uplambda }_{\mathrm{AL}\leftarrow \mathrm{M}}\times {\upsigma }_{\mathrm{u},\mathrm{M}}^{2}$ is the indirect genetic association between ADG_AL_ and ADG_R_ (i.e., the part of the covariance that is due to microbial-mediated effects). The same Eqs. (14), (15), and (16) for the partition of the total genetic variances and covariances also apply to the other variance components of the model.

The SEM was implemented under a Bayesian approach via Gibbs sampling using the Gibbsf90 software of the blupf90 family of programs [27]. Single sampling processes of 1,000,000 iterations were run for all the models, discarding the first 300,000 iterations of each chain and saving 1 of every 100 samples. Posterior marginal inferences on variance components were performed with the Postgibbsf90 software [27]. Structural coefficients were considered to be statistically different from 0 when the high posterior density interval at 95% of confidence level (HPD95%) did not include the 0.

## Results

### Recursive effects

**Fig. 2:**
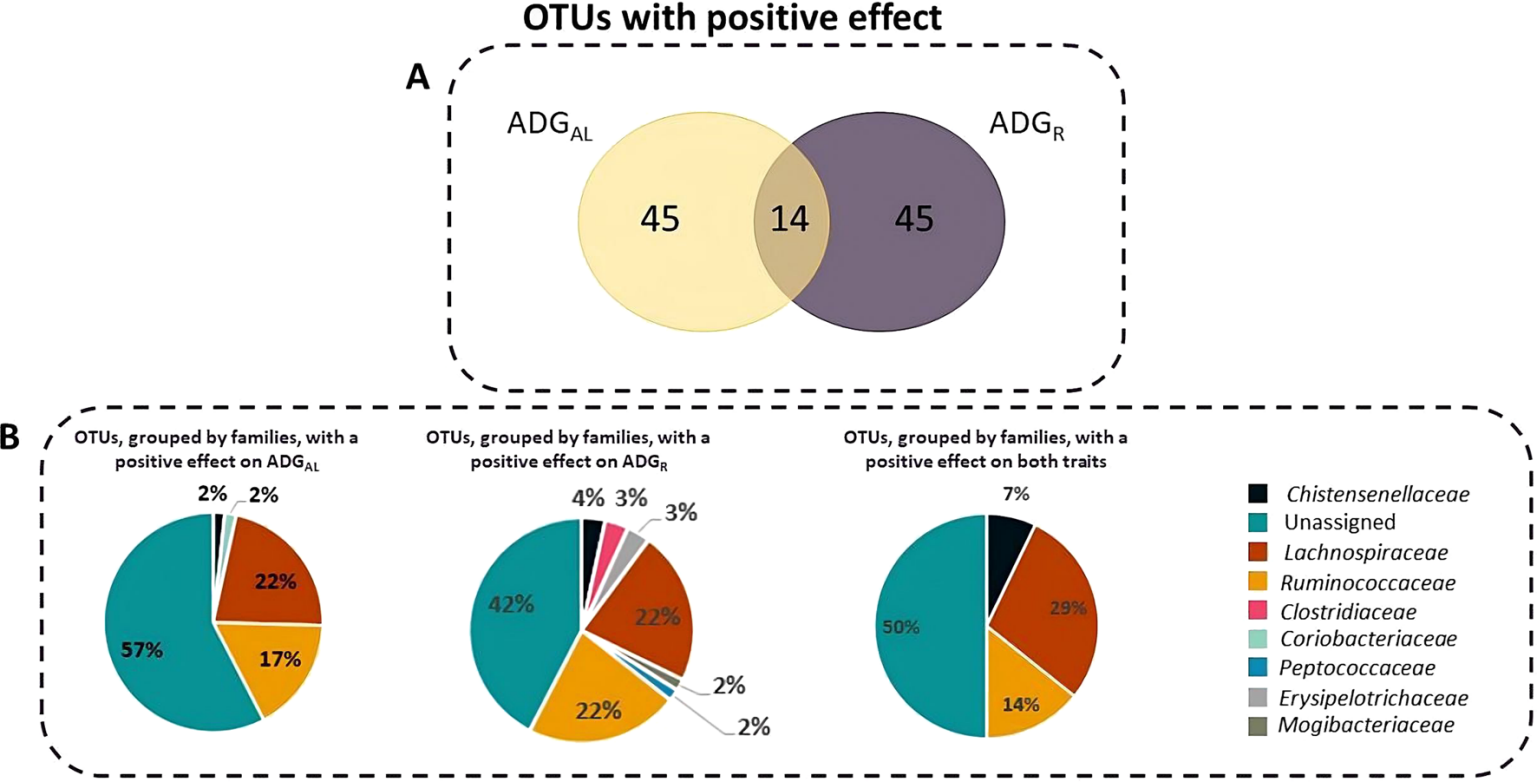
**a** Venn diagram showing the overlap between OTU with an abundance that had a positive effect on average daily gain of growing rabbits fed ad libitum (ADG_AL_) and under restricted feeding (ADG_R_) and **b** distribution of the OTU, grouped by families, with a positive effect on ADG_AL_, on ADG_R_, or on both traits

Our SEM approach allowed the identification of 138 OTU with structural coefficients that were statistically different from 0: 82 OTU had an effect on ADG_AL_, 80 on ADG_R_, and 24 on both traits. Of these 138 OTU, 104 had a positive effect, with 45 affecting ADG_AL_, 45 affecting ADG_R_, and 14 affecting both traits. Note that the resolution of the 16S rDNA locus that was used in this study to characterize the cecal microbiota allowed annotation of only about half of all sequences at the family level. Thus, most of the OTU with a positive effect on ADG could not be assigned at the family level (57, 42, and 50% for ADG_AL_, ADG_R_, and both traits, respectively). In the three cases, the majority of these OTU belong to the *Lachnospiraceae* and *Ruminococcaceae* families. For these two families, 22 and 17%, respectively, of the total number of OTU had a positive effect on ADG_AL_, 22 and 22% on ADG_R_, and 29 and 14% on both traits (Fig. 2).

**Fig. 3:**
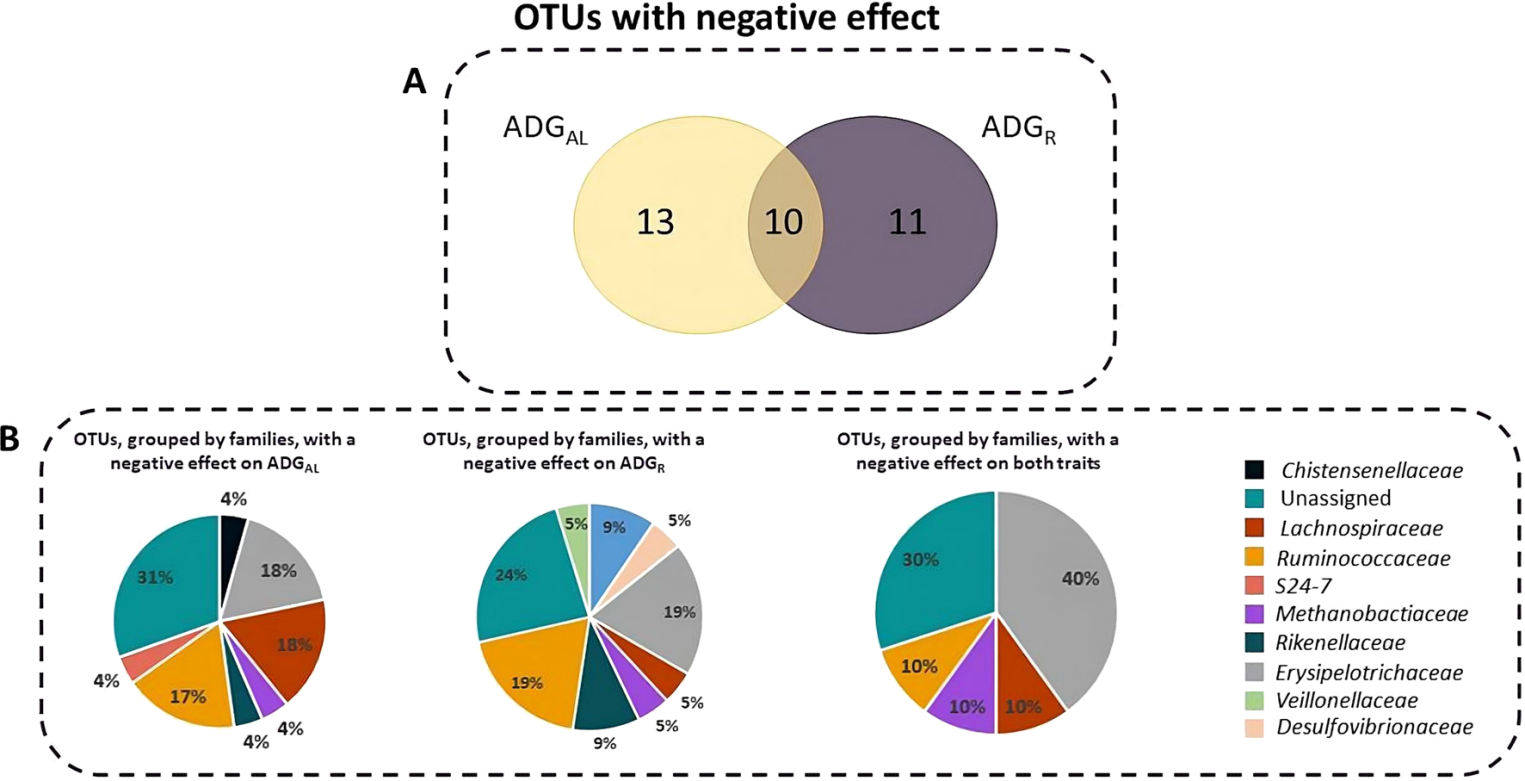
**a** Venn diagram showing the overlap between the OTU with a an abundance that had a negative effect on average daily gain of growing rabbits fed ad libitum (ADG_AL_) and under restricted feeding (ADG_R_) and **b** distribution of the OTU, grouped by families, with a negative effect on ADG_AL_, on ADG_R_ and on both traits

Of the OTU with negative effects, 13 affected ADG_AL_, 11 affected ADG_R_, and 10 affected both traits, of which 31, 24, and 30%, respectively, were not assigned at the family level. Most of these OTU are encompassed by the *Lachnospiraceae* (18%), *Erysipelotricaceae* (18%) and *Ruminococcaceae* families (17%) for ADG_AL_, by the *Ruminococcaceae* (19%) and *Erysipelotricaceae* families (19%) for ADG_R_, and by the *Erysipelotricaceae* family (40%) for both traits (Fig. 3).

**Fig. 4:**
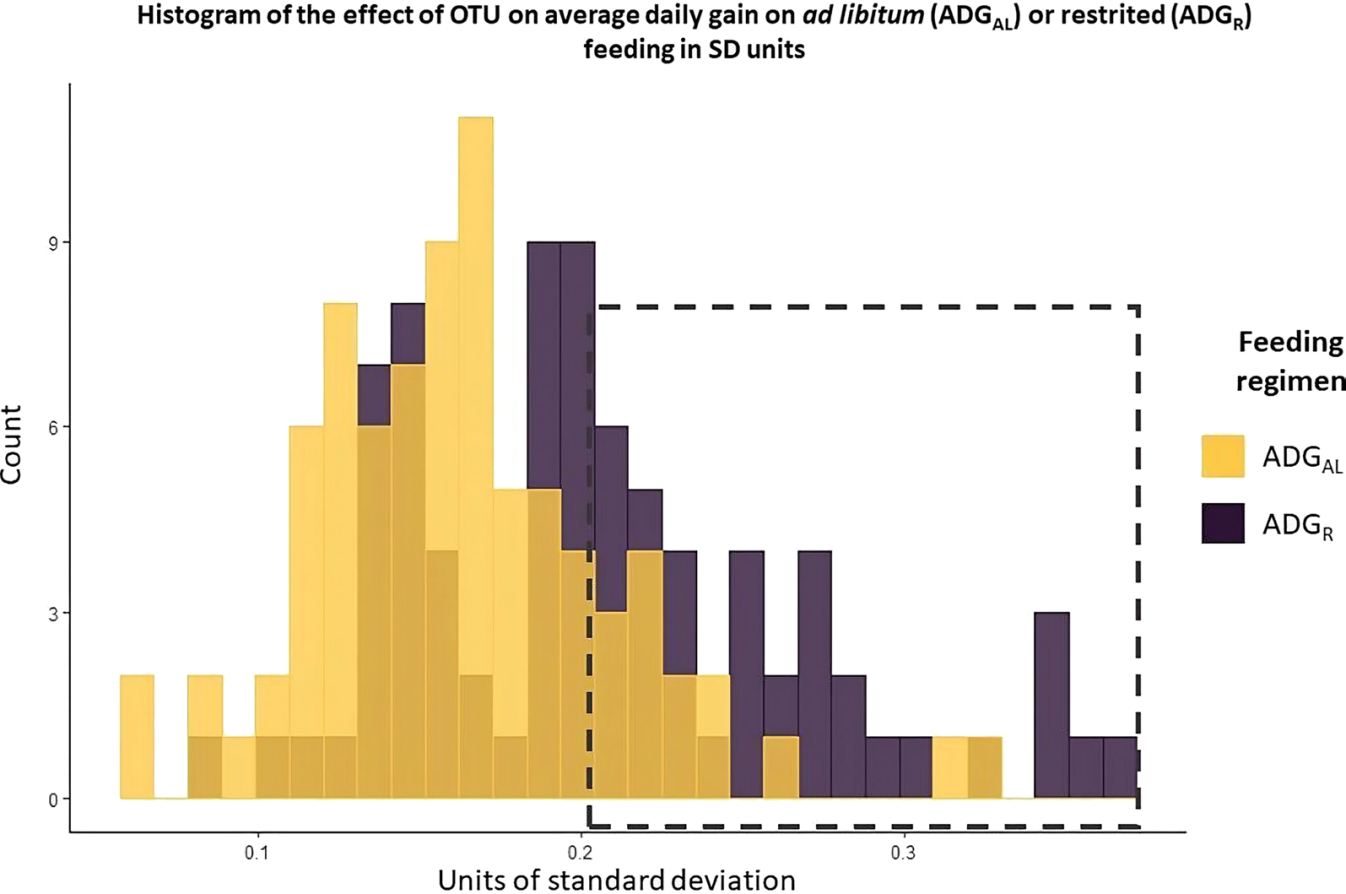
Histogram of the estimates of the effect of abundance of OTU on average daily gain of rabbits fed ad libitum (ADG_AL_) or under restricted feeding (ADG_R_) in SD units of the trait

The marginal posterior means of the positive structural coefficients for the effect of M (i.e. the specific OTU) on ADG_AL_ ranged from 0.369 to 1.271 (g/d CSS-normalized OTU units) (see Additional file 1: Table S1), and from 0.452 to 1.859 (g/d CSS-normalized OTU units) for the effect of M on ADG_R_ (see Additional file 1: Table S2). In turn, the marginal posterior means of the negative structural coefficients for the effect of M on ADG_AL_ ranged from − 0.770 to − 1.907 (g/d CSS-normalized OTU units) (see Additional file 1: Table S1), and from − 0.707 to − 1.929 (g/d CSS-normalized OTU units) for the effect of M on ADG_R_ (see Additional file 1: Table S2). To identify and compare OTU with a substantial effect on ADG_AL_ and ADG_R_, the structural coefficient estimate for each OTU and trait was expressed as the expected change in number of phenotypic standard deviations (SD) for ADG for a one-unit increase in CSS-normalized OTU units by dividing the structural coefficient by the SD of the trait (Fig. 4).

Given the large number of OTU with structural coefficients that were statistically different from 0, only those with an absolute effect equal or higher than 0.2 SD units are described in detail. Estimates of these OTU, which we will refer to as relevant OTU, are listed in Table 2 (for ADG_AL_) and 3 (for ADG_R_). Among the OTU with a negative effect on the production traits, the New.ReferenceOTU4438, a member of the *S24-7* family, was the OTU with the largest effect on ADG_AL_ (− 1.907 CSS-normalized OTU units) (Table 2) and the New.ReferenceOTU369, taxonomically assigned to the *Desulfovibrio* genus, was the OTU with the largest effect on ADG_R_ (− 1.929, g/d, CSS-normalized OTU units) (Table 3). Among the OTU with a positive effect on ADG_R_, a member of the *Ruminococcaceaae* family (New.ReferenceOTU1337) had the largest effect, with an estimated increment of 1.8 g/d per CSS-normalized OTU unit. Of the relevant OTU with a positive effect on ADG_AL_, OTU 641783 that had the largest effect and also belonged to the *Ruminococcaceaae* family, with an estimated increment of 1.3 g/d per CSS-normalized OTU unit. None of the relevant OTU had opposite effects on the two traits. Of the 24 OTU with an effect on both traits, five (21%) were among the relevant OTU and all of these had a negative effect on both traits: New.ReferenceOTU2572 (genus *Coprobacillus*,) New.ReferenceOTU3820 (order *Clostridiales*), New.ReferenceOTU4568 (family *Lachnospiraceae*), 522,353 (genus *Coprobacillus*) and 860,192 (genus *Coprobacillus*).

**Table 2:** Posterior means, posterior medians, and 95% highest posterior density intervals (HPD_95%_) of structural coefficients (in g/d, CSS-normalized OTU units) for the relevant OTU on average daily gain of rabbits fed ad libitum, along with their posterior means expressed in SD units and their assignment at the lowest taxonomic level

OTU	Mean	Median	HPD_95%_	Effect	Taxonomy
New.ReferenceOTU4438	− 1.907	− 1.890	[− 3.488, − 0.393]	− 0.322	Family *S24-7*
209947	− 1.867	− 1.844	[− 3.478, − 0.352]	− 0.315	Order *Clostridiales*
New.ReferenceOTU4568	− 1.537	− 1.546	[− 2.862, − 0.328]	− 0.260	Family *Lachnospiraceae*
339336	− 1.440	− 1.439	[− 2.456, − 0.341]	− 0.244	Order *Clostridiales*
New.ReferenceOTU3820	− 1.438	− 1.435	[− 2.344, − 0.577]	− 0.243	Order *Clostridiales*
860192	− 1.389	− 1.389	[− 2.355, − 0.452]	− 0.235	Genus *Coprobacillus*
522353	− 1.355	− 1.351	[− 2.312, − 0.375]	− 0.229	Genus *Coprobacillus*
New.ReferenceOTU2572	− 1.327	− 1.322	[− 2.300, − 0.455]	− 0.224	Genus *Coprobacillus*
New.ReferenceOTU1080	− 1.310	− 1.312	[− 2.093, − 0.548]	− 0.221	Genus *Blaultia*
641783	1.276	1.276	[0.367, 2.219]	0.215	Family *Ruminococcae*
New.ReferenceOTU782	1.271	1.268	[0.256, 2.315]	0.215	Unassigned
New.ReferenceOTU776	1.252	1.249	[0.148, 2.276]	0.212	Family *Lachnospiraceae*
554303	− 1.245	− 1.244	[− 2.055, − 0.476]	− 0.211	Family *Lachnospiraceae*
New.ReferenceOTU2945	1.210	1.203	[0.452, 2.030]	0.205	Unassigned
New.ReferenceOTU3271	1.194	1.195	[0.055, 2.461]	0.202	Order *Clostridiales*

**Table 3:** Posterior means, posterior medians, and 95% highest posterior density intervals (HPD_95%_) of structural coefficients (in g/d, CSS-normalized OTU units) for the relevant OTU on average daily gain of rabbits fed on restriction*,* along with their posterior means expressed in SD units and their assignment at the lowest taxonomic level

OTU	Mean	Median	HPD_95%_	Effect	Taxonomy
New.ReferenceOTU369	− 1.929	− 1.904	[− 3.224, − 0.699]	− 0.366	Genus *Desulfovibrio*
New.ReferenceOTU1337	1.859	1.871	[0.158, 3.403]	0.353	Family *Ruminococcaceae*
New.ReferenceOTU381	1.838	1.858	[0.110, 3.435]	0.349	Family *Lachnospiraceae*
New.ReferenceOTU1863	1.833	1.837	[0.857, 2.829]	0.348	Order *Clostridiales*
207340	1.793	1.791	[0.573, 3.018]	0.340	Family *Mogibacteriaceae*
New.ReferenceOTU570	− 1.704	− 1.624	[− 3.194, − 0.475]	− 0.323	Genus *Phascolarctobacterium*
New.ReferenceOTU3941	1.611	1.598	[0.745, 2.548]	0.306	Family *Christensenellaceae*
New.ReferenceOTU2872	1.546	1.536	[0.659, 2.449]	0.293	Order *Clostridiales*
213671	− 1.486	− 1.488	[− 2.559, − 0.290]	− 0.282	Family *Rikenellaceae*
New.ReferenceOTU3611	− 1.481	− 1.476	[− 2.675, − 0.233]	− 0.281	Family *Erysipelotrichaceae*
New.ReferenceOTU3526	1.447	1.443	[0.624, 2.253]	0.275	Family *Lachnospiraceae*
522353	− 1.438	− 1.439	[− 2.494, − 0.406]	− 0.273	Genus *Coprobacillus*
New.ReferenceOTU4568	− 1.428	− 1.408	[− 2.572, − 0.352]	− 0.271	Family *Lachnospiraceae*
644244	− 1.423	− 1.421	[− 2.532, − 0.394]	− 0.270	Order *Clostridiales*
New.ReferenceOTU1522	1.400	1.404	[0.168, 2.561]	0.266	Family *Ruminococcaceae*
New.ReferenceOTU3977	1.352	1.305	[0.498, 2.223]	0.257	Family *Lachnospiraceae*
New.ReferenceOTU413	1.340	1.327	[0.275, 2.315]	0.254	Family *Ruminococcaceae*
348609	1.317	1.309	[0.493, 2.123]	0.250	Family *Christensenellaceae*
211066	− 1.314	− 1.310	[− 2.396, − 0.189]	− 0.249	Family *Ruminococcaceae*
860192	− 1.301	− 1.297	[− 2.370, − 0.293]	0.247	Genus *Coprobacillus*
New.ReferenceOTU4280	1.269	1.247	[0.044, 2.469]	0.241	Species *Gnavus*
New.ReferenceOTU3816	1.216	1.217	[0.051, 2.323]	0.231	Family *Ruminococcaceae*
New.ReferenceOTU3320	1.203	1.209	[0.376, 1.950]	0.228	Order *Clostridiales*
New.ReferenceOTU2572	− 1.194	− 1.201	[− 2.156, − 0.246]	− 0.227	Genus *Coprobacillus*
New.ReferenceOTU3360	1.189	1.203	[0.269, 2.046]	0.226	Order *Clostridiales*
New.ReferenceOTU3820	− 1.186	− 1.181	[− 2.297, − 0.016]	− 0.225	Order *Clostridiales*
New.ReferenceOTU1988	1.173	1.173	[0.254, 2.059]	0.223	Order *Clostridiales*
New.ReferenceOTU362	1.165	1.140	[0.026, 2.341]	0.221	Family *Ruminococcaceae*
New.ReferenceOTU1139	1.161	1.165	[0.123, 2.222]	0.220	Family *Lachnospiraceae*
New.ReferenceOTU4624	− 1.145	− 1.147	[− 1.969, − 0.325]	− 0.217	Family *Ruminococcaceae*
New.ReferenceOTU669	− 1.125	− 1.135	[− 2.059, − 0.125]	− 0.213	Family *Ruminococcaceae*
266198	1.108	1.113	[0.031, 2.120]	0.210	Order *RF39*
New.ReferenceOTU591	1.102	1.107	[0.174, 1.971]	0.209	Family *Peptococcaceae*
1108356	1.097	1.096	[0.195, 2.048]	0.208	Order *RF39*
New.ReferenceOTU2960	− 1.093	− 1.0.94	[− 1.780, − 0.433]	− 0.207	Order *Clostridiales*
New.ReferenceOTU4631	1.093	1.106	[0.123, 2.094]	0.207	Order *Clostridiales*
New.ReferenceOTU1502	1.066	0.068	[0.322, 1.717]	0.202	Genus *Blautia*
New.ReferenceOTU1728	1.055	1.067	[0.098, 2.060]	0.200	Genus *Coprococcus*

### Contribution of direct and microbial-mediated genetic effects to the genetic variance

**Fig. 5:**
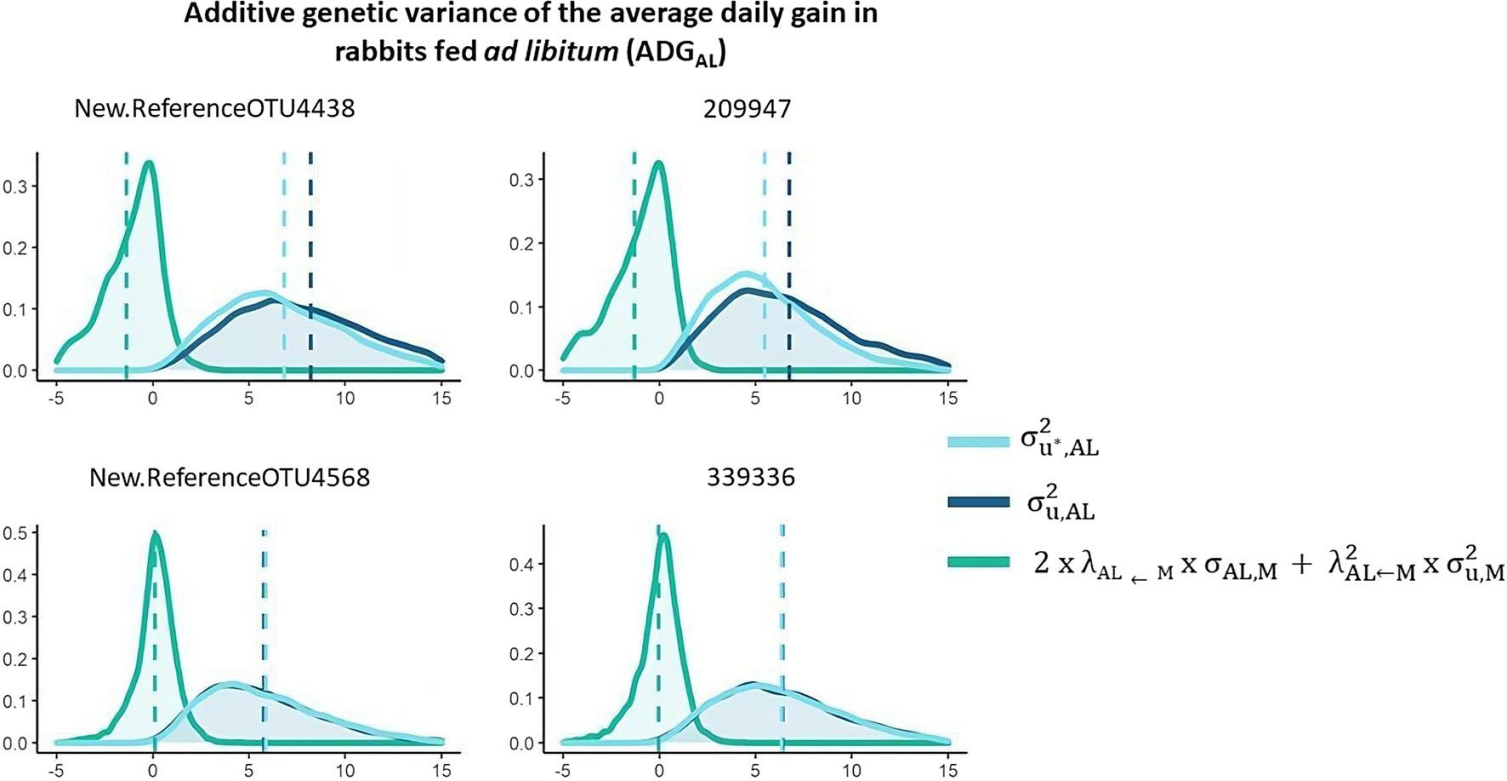
Estimates of the total additive genetic variance (${\upsigma }_{{\mathrm{u}}^{*},\mathrm{AL}}^{2}$) for average daily gain of rabbits fed ad libitum (ADG_AL_), and of direct (${\upsigma }_{{\mathrm{u}},\mathrm{AL}}^{2}$) and indirect genetic effects (${2\times\uplambda }_{\mathrm{AL}\leftarrow \mathrm{M}}\times {\upsigma }_{\mathrm{u},\mathrm{M},\mathrm{AL}}+{\uplambda }_{\mathrm{AL}\leftarrow \mathrm{M}}^{2}\times {\upsigma }_{\mathrm{u},\mathrm{M}}^{2}$) that contribute to this variance for the most relevant OTU

**Fig. 6:**
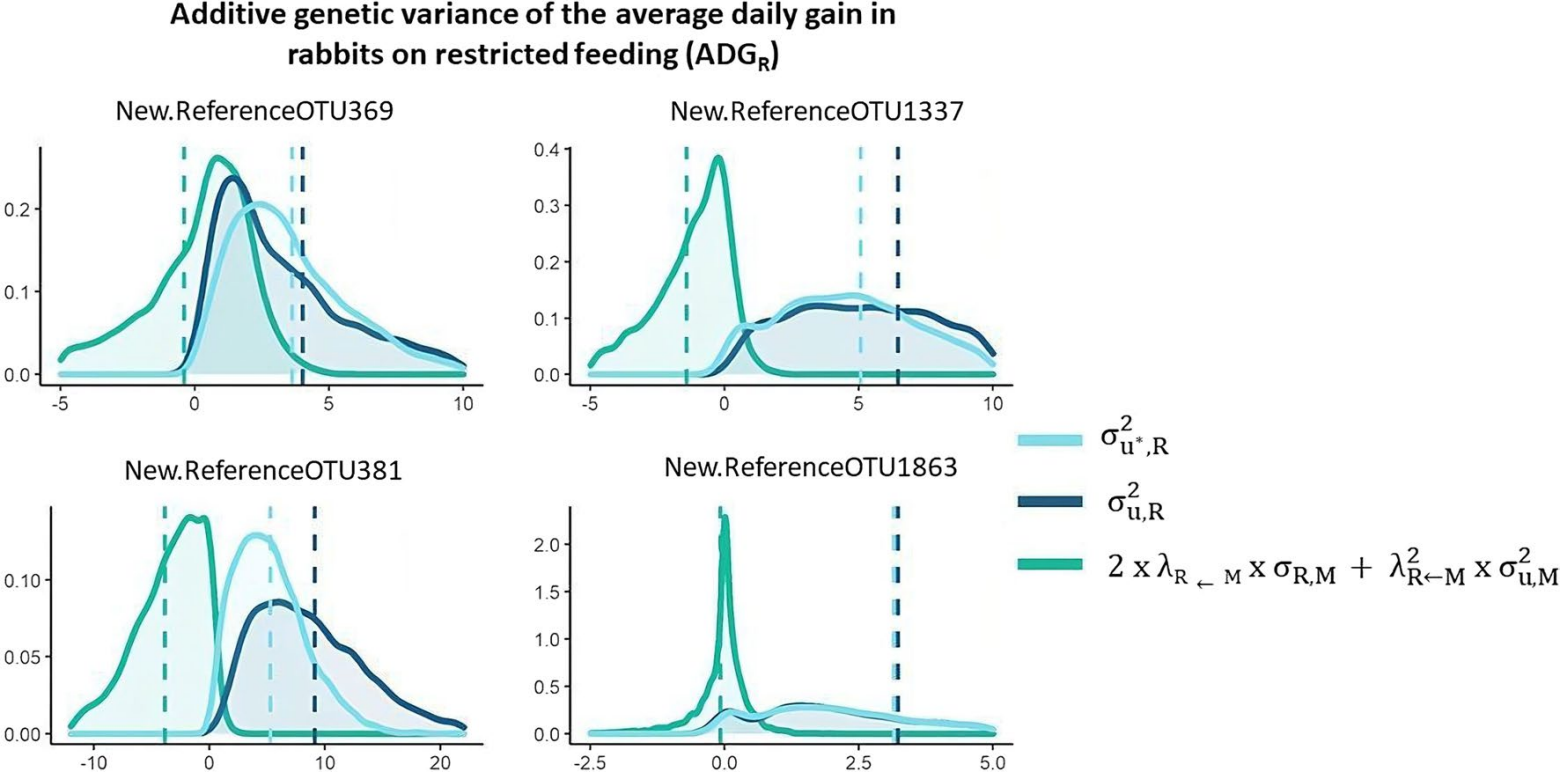
Estimates of the total additive genetic variance (${\upsigma }_{{\mathrm{u}}^{*},\mathrm{R}}^{2}$) of the average daily gain of rabbits under restricted feeding (ADG_R_), and of direct effects (${\upsigma }_{{\mathrm{u}},\mathrm{R}}^{2}$) and indirect effects (${2\times\uplambda }_{\mathrm{R}\leftarrow \mathrm{M}}\times {\upsigma }_{\mathrm{u},\mathrm{M},\mathrm{R}}+{\uplambda }_{\mathrm{R}\leftarrow \mathrm{M}}^{2}\times {\upsigma }_{\mathrm{u},\mathrm{M}}^{2}$) that contribute to this variance of the most relevant OTU

Figures 5 and 6 show the marginal posterior distribution of the total genetic variances (${\upsigma }_{{\mathrm{u}}^{*},\mathrm{AL}}^{2}$ and ${\upsigma }_{{\mathrm{u}}^{*},\mathrm{R}}^{2}$ for ADG_AL_ and ADG_R_, respectively) and of the genetic variances of direct (${\upsigma }_{\mathrm{u},\mathrm{AL}}^{2}$ and ${\upsigma }_{\mathrm{u},\mathrm{R}}^{2}$ for ADG_AL_ and ADG_R_, respectively) and indirect effects (${2\times\uplambda }_{\mathrm{AL}\leftarrow \mathrm{M}}\times {\upsigma }_{\mathrm{u},\mathrm{M},\mathrm{AL}}+{\uplambda }_{\mathrm{AL}\leftarrow \mathrm{M}}^{2}\times {\upsigma }_{\mathrm{u},\mathrm{M}}^{2}$ and ${2\times\uplambda }_{\mathrm{R}\leftarrow \mathrm{M}}\times {\upsigma }_{\mathrm{u},\mathrm{M},\mathrm{R}}+{\uplambda }_{\mathrm{R}\leftarrow \mathrm{M}}^{2}\times {\upsigma }_{\mathrm{u},\mathrm{M}}^{2}$ for ADG_AL_ and ADG_R_, respectively) for the most relevant OTU regarding their effects on ADG_AL_ (Fig. 5) and ADG_R_ (Fig. 6). In general, the distributions of the total and the direct genetic variances were nearly the same because of the small contribution of indirect effects. However, two relevant OTU of the *Clostridiales* order (New.ReferenceOTU1337 and New.ReferenceOTU381 belonging to the *Ruminococcaceae* and *Lachnospiraceae* families, respectively) exerted a negative indirect effect on ADG_R_ (Fig. 6), while OTU 209947, also of the *Clostridiales* order, negatively affected ADG_AL_. OTU that belong to other orders also affected ADG_AL_ (New.ReferenceOTU4438, *Bacteroidales* order) and ADG_R_ (New.ReferenceOTU369, *Desulfovibrionales* order), but the contributions of their indirect effects to the total genetic variance were smaller.

### Heritability estimates for ADG and the relevant OTU

**Fig. 7:**
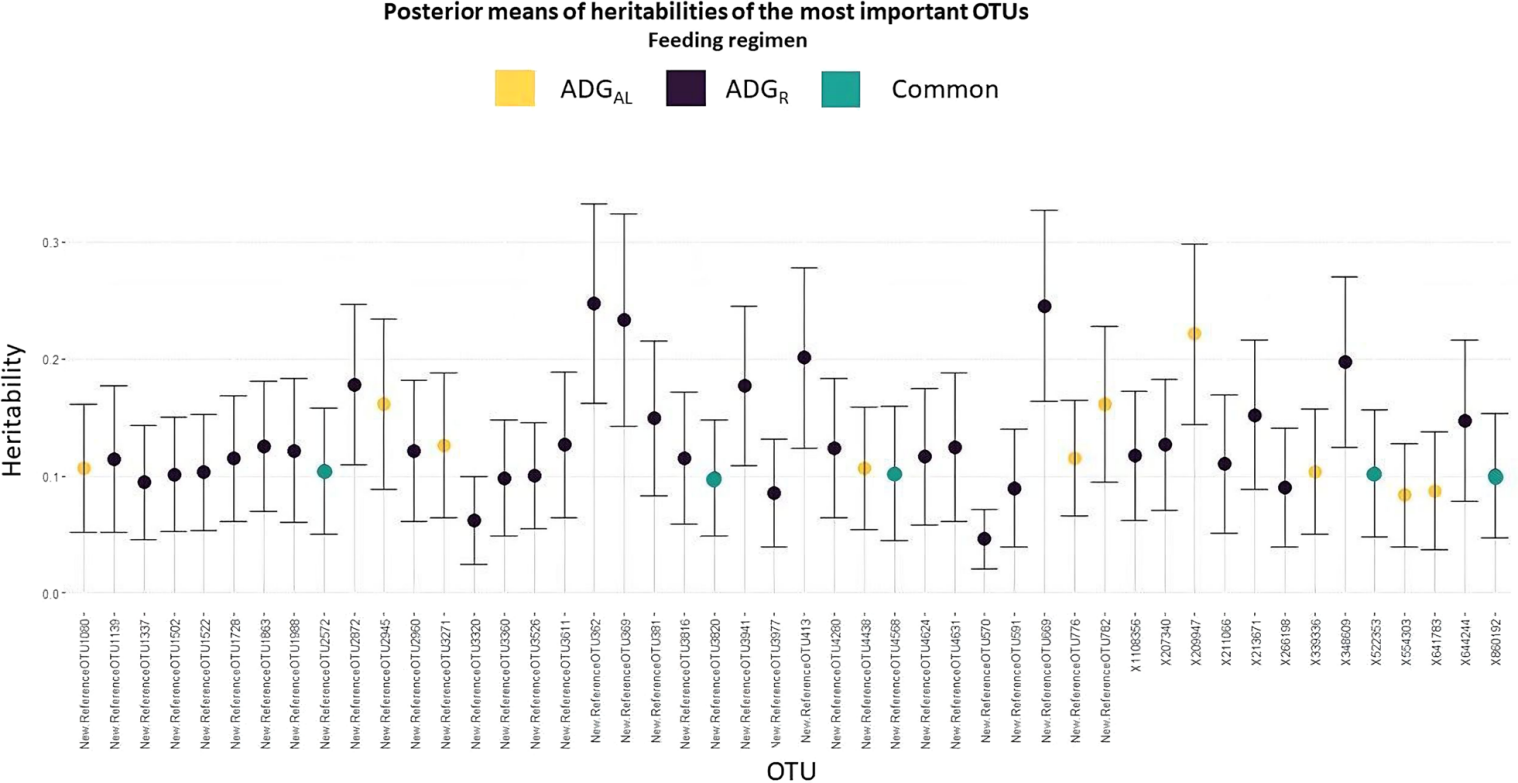
Posterior means of heritabilities of the abundance of OTU that have an important effect on growth rate of rabbits fed ad libitum (ADG_AL_), on rabbits on restricted feeding (ADG_R_) or in both feeding regimes (common)

Average heritability estimates across all 946 analyses for the two ADG traits were low to moderate, with posterior means (SD) of 0.215 (0.104) and 0.157 (0.101) for ADG_AL_ and ADG_R_, respectively. Means (SD) of the marginal posterior distributions for the heritabilities for the most relevant OTU that affected ADG_AL_, and ADG_R_, or both, are shown in Fig. 7. In general, the estimates of these heritabilities were also low to moderate. Posterior mean heritabilities for the relevant OTU for ADG_AL_ ranged from 0.083 for OTU 522353 to 0.222 for OTU 209947, both members of the *Clostridiales* order. Posterior mean heritability estimates for the relevant OTU for ADG_R_ ranged from 0.046 for a member of the *Phascolarctobacterium* genus (New.ReferenceOTU570) to 0.234 for the New.ReferenceOTU369, which belongs to the *Desulfovibrio* genus. The most heritable families were *Desulfovibrionaceae* (0.23) and *Methanobacteriaceae* (0.24), while the least heritable families were *Veillonellaceae* (0.04) and *Peptococcaceae* (0.08).

### Direct genetic covariances between ADG and OTU

**Fig. 8:**
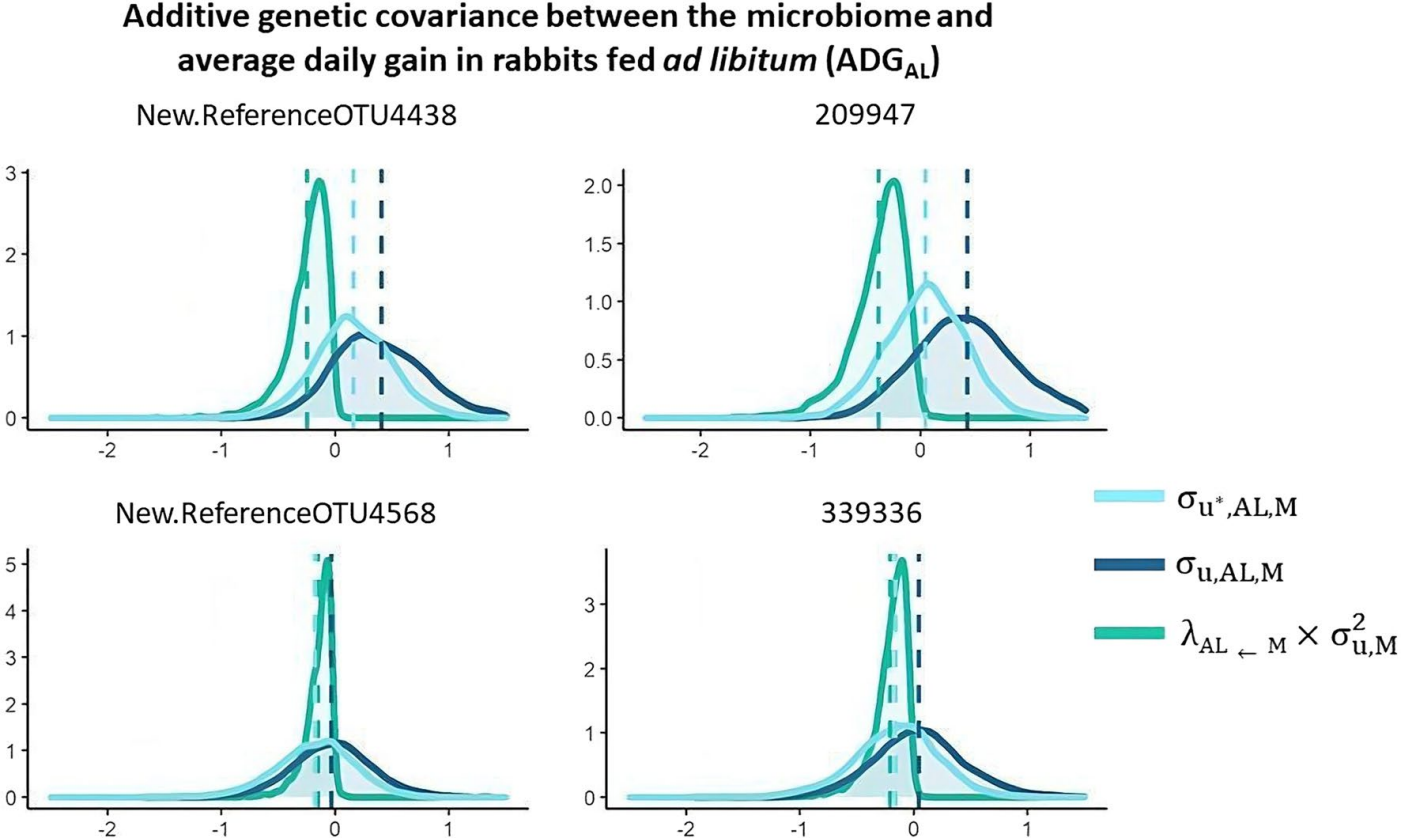
Additive genetic covariance between average daily gain in rabbits fed ad libitum (ADG_AL_) and abundance of the most important OTU: total additive covariance (${\upsigma }_{{\mathrm{u}}^{*},\mathrm{AL},\mathrm{M}}$), direct effects (${\upsigma }_{\mathrm{u},\mathrm{AL},\mathrm{M}}$), and indirect effects (${\uplambda }_{\mathrm{AL}\leftarrow \mathrm{M}}\times {\upsigma }_{\mathrm{u},\mathrm{M}}^{2}$)

**Fig. 9:**
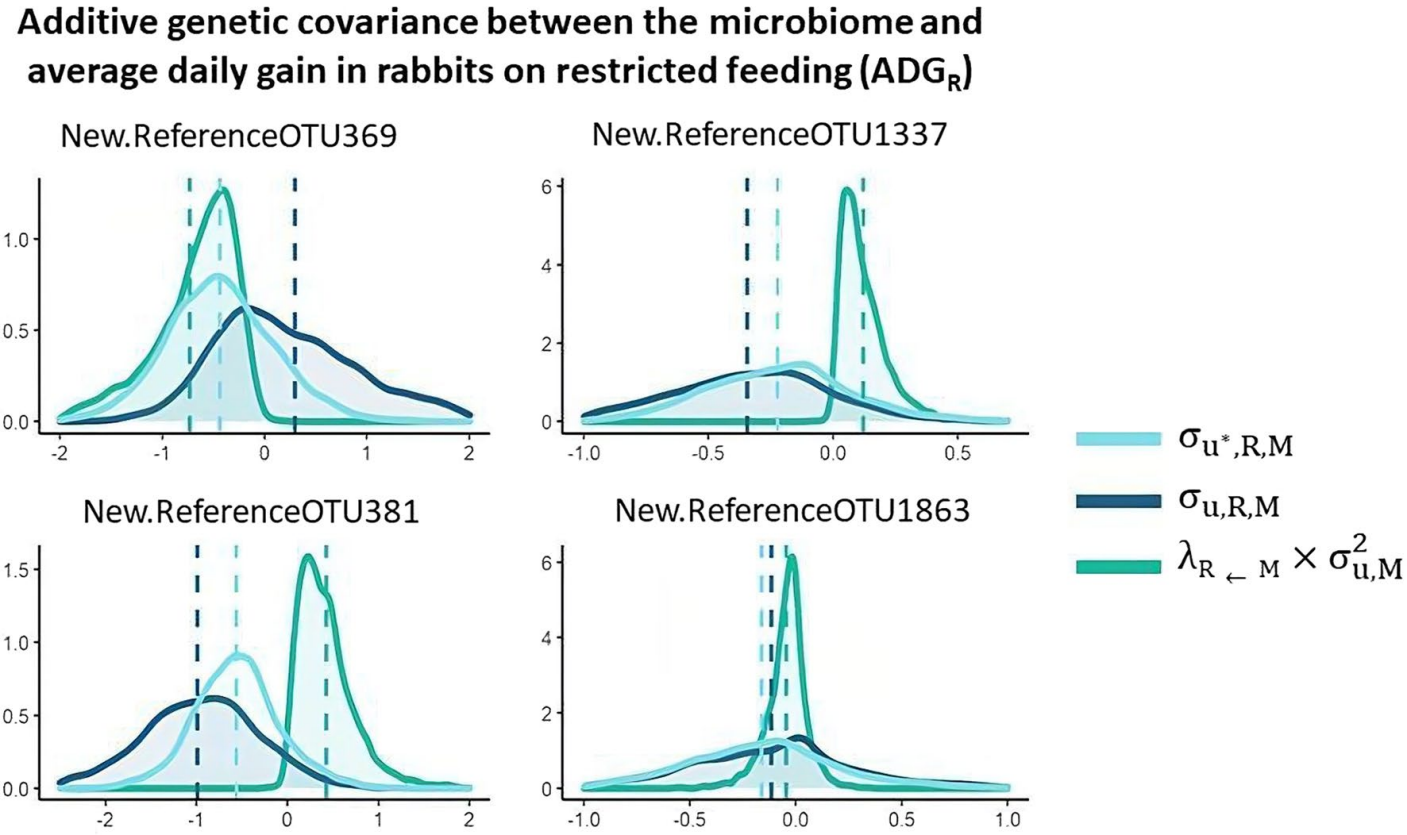
Additive genetic covariance between average daily gain in rabbits under restricted feeding (ADG_R_) and abundance of the most important OTU: total additive covariance (${\upsigma }_{{\mathrm{u}}^{*},\mathrm{R},\mathrm{M}}$), direct effects (${\upsigma }_{\mathrm{u},\mathrm{R},\mathrm{M}}$) and indirect effects (${\uplambda }_{\mathrm{R}\leftarrow \mathrm{M}}\times {\upsigma }_{\mathrm{u},\mathrm{M}}^{2}$)

Marginal posterior distributions of the total additive genetic covariance between ADG_AL_ and M (${\upsigma }_{{\mathrm{u}}^{*},\mathrm{AL},\mathrm{M}}$), of the covariance due to direct effects (${\upsigma }_{\mathrm{u},\mathrm{AL},\mathrm{M}}$), and of the covariance due to indirect effects of M on ADG_AL_ (${\uplambda }_{\mathrm{AL}\leftarrow \mathrm{M}}\times {\upsigma }_{\mathrm{u},\mathrm{M}}^{2}$) are represented in Fig. 8. The same distributions are shown in Fig. 9 for ADG_R_ (${\upsigma }_{{\mathrm{u}}^{*},\mathrm{R},\mathrm{M}}$, ${\upsigma }_{\mathrm{u},\mathrm{R},\mathrm{M}}$ and ${\uplambda }_{\mathrm{R}\leftarrow \mathrm{M}}\times {\upsigma }_{\mathrm{u},\mathrm{M}}^{2}$, for total, direct, and indirect covariances, respectively). Total and direct additive genetic covariances were very similar for the New.ReferenceOTU4568, OTU 339336, and New.ReferenceOTU1863 (all members of the *Clostridiales* order), due to the almost zero indirect effects. The indirect effects of the OTU 209947, which belongs to the *Clostridiales* order, on ADG_AL_ were substantial and negative, while the indirect effects of members of the *Desulfovibrio* genus (New.ReferenceOTU369) and the *Lachnospiraceae* family (New.ReferenceOTU381) on ADG_R_ were substantial and negative and positive, respectively.

## Discussion

In this study, SEM were implemented to assess causal relationships between the cecal microbiota and two traits related to rabbit growth and FE. The effect that a specific OTU exerts on the studied phenotypes represents the indirect effects (i.e. the effect of the OTU on the trait while holding host genetics and environmental effects constant). It is measured by the structural coefficient, which quantifies the expected change in the phenotype by a one-unit increase in CSS-normalized OTU units of a specific OTU. When direct and indirect effects are of the same magnitude but have opposite signs, the total effect on the trait becomes null. In such a scenario, a GWAS can fail to identify the genomic regions that affect the production trait through multiple paths. This hypothesis was tested in a previous study conducted by Tiezzi et al. [28], who evaluated how host genetics can affect fat deposition in pigs by affecting gut microbiome composition, which then results in a change in the phenotype. In that study, it was demonstrated that the genes that do not affect the phenotype directly can be identified in GWAS in which effects mediated by the microbiome were included in the model. This highlights the importance of knowing the direct and indirect effects on a phenotype when designing sustainable strategies to improve productivity and sustainability in livestock. To our knowledge, the importance of direct versus indirect effects on growth rate through microbial diversity and composition have not yet been quantified. The only related study was by Saborio et al. [29], who implemented a SEM approach to estimate the effect of the relative abundance of rumen microbes on methane production in dairy cattle.

Most of the OTU that had statistically significant structural coefficients and that positively affected ADG_AL_ and ADG_R_ belong to the *Lachnospiraceae* or *Ruminococcaceae* families. Conversely, the OTU that had statistically significant structural coefficients and that negatively affected ADG_AL_ belong to a wider set of families: *Ruminococcaceae*, *Lachnospiraceae*, *Methanobacteriaceae,* and *Erysipelotrichaceae*. *Lachnospiraceae* and *Ruminococcaceae* are both members of the *Clostridiales* order. Several members of this order have previously been found to be associated with FE across different livestock species, including pigs [30, 31], chickens [32] and beef cattle [33]. *Erysipelotrichaceae* is a family that is associated with lipid metabolism and has been linked to inflammation [34]. However, its increase in abundance has been associated with the most but also with the least efficient pigs, depending on the sampling origin that each study chose for gut microbiota characterization [35].

As previously mentioned, given the large number of OTU with statistically significant structural coefficients that were identified by our SEM approach, our discussion will be focused on the OTU that had a substantial effect (equal to or larger than 0.2 units of SD of the trait) on the phenotype, i.e. the relevant OTU. We identified 15 and 38 OTU with relevant effects on ADG_AL_ and ADG_R_, respectively. The estimated effects for the relevant OTU for ADG_R_ were larger than those for the OTU affecting ADG_AL_. Thus, only 18% of the OTU that affected ADG_AL_ had a structural coefficient larger than 0.2 SD units for ADG_AL_, while 47% of the OTU that affected ADG_R_ met this criterion. This could indicate that growth rate is mainly determined by host genetics whereas FE is determined by both the host genetics and the gut microbiota. This is in agreement with results reported by Velasco-Galilea et al. [36], who found that the predictive value of cecal microbiota was higher for ADG_R_ than for ADG_AL_.

It is worth highlighting the effects of some other OTU on ADG_AL_ or ADG_R_. For instance, a member of the *Phascolarctobacterium* genus (New.ReferenceOTU570) had a substantial effect on ADG_R_ (− 1.70 [− 3.194, − 0.475]). Some species of this genus produce acetate and propionate short-chain fatty acids (SCFA), which act as energy sources. Propionate is a gluconeogenic substrate in the liver and intestine, while acetate contributes to the synthesis of lipids [37, 38] and has been shown to play a direct role in the regulation of appetite in mice [39]. Another OTU with an abundance that had a substantial positive effect on FE was the New.ReferenceOTU381 (*Lachnospiraceae* family). Velasco-Galilea et al. [36] reported five OTU of the *Lachnospiraceae* family (including the New.ReferenceOTU381) to be among the most informative OTU to predict rabbit growth and FE. Two and three of these OTU were, respectively, positively and negatively correlated with average daily residual feed intake in rabbits fed ad libitum.

Genetic variances for ADG_AL_ and ADG_R_ in the SEM can be interpreted as the variance due to direct genetic effects on the traits free from the genetic effects mediated by M that have a causal effect on them. Indirect genetic effects can act by reducing the total genetic variance if they are strong enough and opposite in sign to the genetic covariance between M and the phenotype. This could make it difficult to assess the genetic determinism of a trait affected by M based on MTAM. However, a SEM enables the different sources of genetic variation to be disentangled. In addition, it enables prediction of the effects of external interventions on a set of variables. In our study, an external intervention could involve promoting or blocking the presence of some cecal microbe.

One member of the *Ruminococcaceae* family (New.ReferenceOTU1337) and one member of the *Lachnospiraceae* family (New.Reference381) had relevant effects on the total genetic variance for ADG_R_, with indirect effects that reduced the total variance, although the estimates of the structural coefficients for these OTU were positive. This was due to the negative genetic correlation between ADG_R_ and the abundance of these OTU. It is worth mentioning that some members of the *Lachnospiraceae* family are butyrate-producing bacteria that have a beneficial effect on animal gut health [40] and have been previously identified to have a strong effect on FE traits in pigs [41].

Estimates of total heritability for ADG_AL_ and ADG_R_ are similar to those estimated in previous studies for the same rabbit population, with a posterior mean of 0.21 and 0.08, respectively, by Piles and Sánchez [5] and with a posterior mean of 0.15 and 0.09, respectively, by Velasco-Galilea et al. [36]. Conversely, estimates of direct heritability based on the SEM were slightly greater than estimates of total heritability for both traits. This could be due to the negative effects of host genetics exerted through the microbiota on these growth traits. In general, estimated heritabilities for the OTU with the largest structural coefficients were low to moderate, suggesting a weak host genetic control of the rabbit cecal microbiota, which is in agreement with results of Velasco-Galilea et al. [12], who assessed the host genetics effects on the rabbit cecal microbiota by means of Bayes factors. The highest heritability [0.234 (0.091)] was estimated for New.ReferenceOTU369, which belongs to the *Desulfovibrio* genus and is one of the OTU that had a relevant effect on ADG_R_. This OTU was previously reported as heritable by Velasco-Galilea et al. [12].

In SEM, the genetic covariance reflects the association between direct effects and can be considered to be due to genes that directly affect two traits simultaneously or to linkage disequilibrium between pairs of genes that each affect one of the two traits [13]. However, there is a second source of association between the host phenotype and M because the host genetic effect on M also affects the host phenotype indirectly. This second source of covariation could even have an opposite sign compared to the first source (i.e., the covariance between direct genetic effects). A member of the *Desulfovibrio* genus (New.ReferenceOTU369) impaired ADG_R_, and the total indirect effects that this OTU was estimated to exert on the phenotype acted by reducing the total effects. In this particular case, direct and total covariances were opposite in sign. Thus, the genetic correlation between this particular OTU and ADG_R_ would be positive if only the effects of host genetics were considered. On the contrary, when the total effects are considered, the relevant negative effect of the *Desulfovibrio* genus on ADG_R_ can be captured by the negative genetic correlation between the OTU and growth. This result is in agreement with previous reports in pigs [30, 42] in which the abundances of members of the *Desulfovibrio* genus were estimated to be negatively correlated with FE.

## Conclusions

Our study highlights the importance of knowing the direct effects that host genetics exerts on a phenotype, as well as the indirect effects that host genetics exerts through the gut microbiota, not only to fully describe the processes of mediation, but also to understand the change in a phenotype that can result from a modification of the microbial composition through an external intervention (e.g., by blocking/promoting the presence of a particular microorganism). This is the first study to evaluate the direct and indirect effects exerted by host genetics on growth. Our results show that 63% of the OTU with abundances that had relevant effects on ADG_R_ had positive effects on this trait. Abundance of one member of the *Desulfovibrio* genus exerted the largest negative effect on ADG_R_, followed by abundance of one member of the *Ruminococcaceae* family, which positively affected this trait. In contrast, only 33% of the OTU that had a relevant effect on ADG_AL_ had a positive effect on this trait.

## Supplementary Information

**Additional file 1: Table S1.** Posterior means, posterior medians, 95% highest posterior density intervals (HPD_95%_) structural coefficients different from zero (in g/d, CSS-normalized OTU units) that had an effect on the average daily gain of growing rabbits fed ad libitum and their assignment at the lowest taxonomic level. **Table S2.** Posterior means, posterior medians, 95% highest posterior density intervals (HPD_95%_) structural coefficients different from zero (in g/d, CSS-normalized OTU units) that had an effect on the average daily gain of growing rabbits under restricted feeding and their assignment at the lowest taxonomic level.

## Data Availability

The raw sequence data were deposited in the sequence read archive of NCBI under the BioProject accession number PRJNA524130.
