# Supplementary material for: Disentangling the causal relationship between rabbit growth and cecal microbiota through structural equation models
Source: Genet Sel Evol. 2022 Dec 19;54:81. doi: 10.1186/s12711-022-00770-2 (PMC9762025; doi:10.1186/s12711-022-00770-2)
Supplement: Supplementary file 1 — Additional file 1: Table S1. Posterior means, posterior medians, 95% highest posterior density intervals (HPD95%) structural coefficients different from zero (in g/d, CSS-normalized OTU units) that had an effect on the average daily gain of growing rabbits fed ad libitum and their assignment at the lowest taxonomic level. Table S2. Posterior means, posterior medians, 95% highest posterior density intervals (HPD95%) structural coefficients different from zero (in g/d, CSS-normalized OTU units) that had an effect on the average daily gain of growing rabbits under restricted feeding and their assignment at the lowest taxonomic level. [file 12711_2022_770_MOESM1_ESM.docx]

**Addittional file 1 Table S1**

**Posterior means, posterior mediasn, 95% highest posterior density intervals (HPD_95%_) structural coefficients different from zero (in g/d, CSS-normalized OTU units) that had an effect on the average daily gain of growing rabbits fed *ad libitum* and their assignment at the lowest taxonomic level.**

| **OTU** | **Mean** | **Median** | **HPD_95%_** | **Taxonomy** |
| --- | --- | --- | --- | --- |
| **641783** | 1.272 | 1.276 | [0.366,2.219] | Family *Ruminococcaceae* |
| **New.ReferenceOTU782** | 1.272 | 1.267 | [0.256,2.315] | Unassigned |
| **New.ReferenceOTU776** | 1.252 | 1.249 | [0.147,2.276] | Family *Lachnospiraceae* |
| **New.ReferenceOTU2945** | 1.210 | 1.20 | [0.452,2.029] | Unassigned |
| **New.ReferenceOTU2872** | 1.179 | 1.164 | [0.359,2.042] | Order *Clostridiales* |
| **New.ReferenceOTU4534** | 1.178 | 1.182 | [0.035,2.257] | Family *Ruminococcaceae* |
| **849147** | 1.116 | 1.102 | [0.043,2.269] | Order *Clostridiales* |
| **352489** | 1.109 | 1.109 | [0.072,2.129] | Family *Lachnospiraceae* |
| **857827** | 1.073 | 1.063 | [0.125,2.024] | Order *Bacteroidales* |
| **New.ReferenceOTU4525** | 1.064 | 1.060 | [0.413,1.729] | Order *Clostridiales* |
| **New.ReferenceOTU3360** | 1.023 | 1.017 | [0.156,1.898] | Order *Clostridiales* |
| **New.ReferenceOTU3941** | 1.018 | 1.021 | [0.021,1.949] | Family *Christensenellaceae* |
| **New.ReferenceOTU3301** | 1.016 | 1.010 | [0.166,1.790] | Genus *Adlercreutzia* |
| **New.ReferenceOTU3526** | 1.015 | 1.010 | [0.289,1.700] | Family *Lachnospiraceae* |
| **696563** | 1.010 | 1.014 | [0.081,1.867] | Family *Lachnospiraceae* |
| **New.ReferenceOTU2136** | 1.001 | 0.992 | [0.175,1.788] | Order *Clostridiales* |
| **New.ReferenceOTU3066** | 0.997 | 0.993 | [0.225,1.699] | Order *Clostridiales* |
| **230132** | 0.971 | 0.971 | [0.055,1.845] | Order *RF39* |
| **4343981** | 0.964 | 0.963 | [0.194,1.769] | Order *RF39* |
| **523099** | 0.946 | 0.944 | [0.024,1.866] | Family *Lachnospiraceae* |
| **New.ReferenceOTU4382** | 0.936 | 0.934 | [0.155,1.707] | Genus *Faecalibacterium* |
| **New.ReferenceOTU4308** | 0.935 | 0.937 | [0.170,1.734] | Order *Clostridiales* |
| **New.ReferenceOTU3714** | 0.930 | 0.932 | [0.217,1.663] | Genus *Blautia* |
| **268410** | 0.928 | 0.926 | [0.107,1.736] | Order *Clostridiales* |
| **New.ReferenceOTU2035** | 0.927 | 0.921 | [0.051,1.878] | Order *Clostridiales* |
| **New.ReferenceOTU1502** | 0.905 | 0.901 | [0.264,1.577] | Genus *Blautia* |
| **New.ReferenceOTU2377** | 0.905 | 0.901 | [0.044,1.791] | Family *Ruminococcaceae* |
| **New.ReferenceOTU174** | 0.884 | 0.882 | [0.141,1.645] | Genus *Oscillospira* |
| **New.ReferenceOTU3516** | 0.875 | 0.871 | [0.305,1.455] | Family *Lachnospiraceae* |
| **New.ReferenceOTU3977** | 0.864 | 0.861 | [0.131,1.573] | Family *Lachnospiraceae* |
| **313834** | 0.863 | 0.857 | [0.111,1.668] | Order *Clostridiales* |
| **621649** | 0.854 | 0.857 | [0.049,1.649] | Order *Clostridiales* |
| **New.ReferenceOTU1136** | 0.835 | 0.834 | [0.107,1.647] | Family *Lachnospiraceae* |
| **New.ReferenceOTU3060** | 0.828 | 0.831 | [0.155,1.539] | Order *Clostridiales* |
| **New.ReferenceOTU1713** | 0.820 | 0.817 | [0.157,1.522] | Order *Clostridiales* |
| **New.ReferenceOTU1755** | 0.810 | 0.812 | [0.102,1.529] | Order *RF39* |
| **825939** | 0.807 | 0.804 | [0.243,1.381] | Order *Clostridiales* |
| **New.ReferenceOTU1490** | 0.790 | 0.790 | [0.139,1.430] | Family *Ruminococcaceae* |
| **New.ReferenceOTU3143** | 0.769 | 0.758 | [0.023,1.485] | Order *Clostridiales* |
| **213084** | 0.768 | 0.763 | [0.008,1.560] | Family *Ruminococcaceae* |
| **346794** | 0.758 | 0.764 | [0.106,1.375] | Order *Clostridiales* |
| **New.ReferenceOTU1628** | 0.749 | 0.748 | [0.214,1.272] | Family *Ruminococcaceae* |
| **New.ReferenceOTU892** | 0.745 | 0.749 | [0.007,1.420] | Order *Clostridiales* |
| **208042** | 0.724 | 0.716 | [0.046,1.536] | Order *Clostridiales* |
| **New.ReferenceOTU251** | 0.713 | 0.711 | [0.099,1.282] | Genus *Coprococcus* |
| **New.ReferenceOTU3779** | 0.696 | 0.694 | [0.097,1.133] | Order *Clostridiales* |
| **New.ReferenceOTU145** | 0.692 | 0.694 | [0.189,1.175] | Genus *Blautia* |
| **New.ReferenceOTU3833** | 0.691 | 0.691 | [0.024,1.369] | Family *Lachnospiraceae* |
| **New.ReferenceOTU1346** | 0.687 | 0.689 | [0.059,1.257] | Genus *Blautia* |
| **279179** | 0.683 | 0.680 | [0.028,1.349] | Order *RF39* |
| **New.ReferenceOTU2585** | 0.663 | 0.657 | [0.171,1.192] | Family *Ruminococcaceae* |
| **New.ReferenceOTU19** | 0.621 | 0.625 | [0.006,1.213] | Family *Lachnospiraceae* |
| **573287** | 0.614 | 0.608 | [0.023,1.99] | Order *Clostridiales* |
| **New.ReferenceOTU1644** | 0.586 | 0.587 | [0.070,1.090] | Order *RF39* |
| **New.ReferenceOTU2543** | 0.516 | 0.518 | [0.016,1.026] | Order *Clostridiales* |
| **342543** | 0.487 | 0.484 | [0.002,0.979] | Family *Ruminococcaceae* |
| **353339** | 0.390 | 0.390 | [0.011,0.776] | Order *Clostridiales* |
| **112931** | 0.369 | 0.370 | [0,0.747] | Order *Clostridiales* |
| **New.ReferenceOTU4338** | -0.770 | -0.762 | [-1.491,-0.021] | Genus *Rikenella* |
| **New.ReferenceOTU2960** | -0.800 | -0.795 | [-1.368,-0.021] | Order *Clostridiales* |
| **New.ReferenceOTU1122** | -0.843 | -0.837 | [-1.688,-0.040] | Genus *Ruminococcus* |
| **578960** | -0.919 | -0.924 | [-1.588,-0.241] | Family *Lachnospiraceae* |
| **336627** | -0.961 | -0.958 | [-1.814,-0.065] | Order *Clostridiales* |
| **New.ReferenceOTU4624** | -0.972 | -0.973 | [-1.717,-0.267] | Family *Ruminococcaceae* |
| **New.ReferenceOTU4000** | -0.974 | -0.980 | [-1.724,-0.164] | Order *Clostridiales* |
| **New.ReferenceOTU669** | -1.024 | -1.019 | [-2.036,-0.061] | Genus *Methanobrevibacter* |
| **New.ReferenceOTU243** | -1.070 | -1.072 | [-1.863,-0.216] | Family *Ruminococcaceae* |
| **356011** | -1.094 | -1.101 | [-1.721,-0.434] | Genus *Ruminococcus* |
| **New.ReferenceOTU3611** | -1.135 | -1.131 | [-2.343,-0.095] | Family *Erysipelotrichaceae* |
| **537548** | -1.1.39 | -1.139 | [-1.876,-0.461] | Order *Clostridiales* |
| **New.ReferenceOTU1683** | -1.160 | -1.155 | [-2.205,-0.131] | Family *Christensenellaceae* |
| **554303** | -1.245 | -1.244 | [-2.055,-0.476] | Family *Lachnospiraceae* |
| **New.ReferenceOTU1080** | -1.311 | -1.312 | [-2.093,-0.548] | Genus *Blautia* |
| **New.ReferenceOTU2572** | -1.326 | -1.322 | [-2.300,-0.455] | Genus *Coprobacillus* |
| **522353** | -1.355 | -1.355 | [-2.317,-0.375] | Genus *Coprobacillus* |
| **860192** | -1.389 | -1.389 | [-2.355,-0.452] | Genus *Coprobacillus* |
| **New.ReferenceOTU3820** | -1.438 | -1.436 | [-2.344,-0.576] | Order *Clostridiales* |
| **339336** | -1.440 | -1.438 | [-2.459,-0.340] | Order *Clostridiales* |
| **New.ReferenceOTU4568** | -1.536 | -1.545 | [-2.862,-0.327] | Family *Lachnospiraceae* |
| **209947** | -1.866 | -1.844 | [-3.477,-0.352] | Order *Clostridiales* |
| **New.ReferenceOTU4438** | -1.906 | -1.889 | [-3.487,-0.393] | Family *S24-7* |

**Additional file Table S2**

**Posterior means, posterior mediasn, 95% highest posterior density intervals (HPD_95%_) structural coefficients different from zero (in g/d, CSS-normalized OTU units) that had an effect on the average daily gain of growing rabbits under restricted feeding and their assignment at the lowest taxonomic level.**

| **OTU** | **Mean** | **Median** | **HPD_95%_** | **Taxonomy** |
| --- | --- | --- | --- | --- |
| **New.ReferenceOTU1337** | 1.859 | 1.871 | [0.158,3.403] | Family *Ruminococcaceae* |
| **New.ReferenceOTU381** | 1.838 | 1.858 | [0.110,3.435] | Family *Lachnospiraceae* |
| **New.ReferenceOTU1863** | 1.833 | 1.837 | [0.857,2.829] | Order *Clostridiales* |
| **207340** | 1.793 | 1.791 | [0.573,3.018] | Family *Mogibacteriaceae* |
| **New.ReferenceOTU3941** | 1.611 | 1.598 | [0.745,2.548] | Family *Christensenellaceae* |
| **New.ReferenceOTU2872** | 1.546 | 1.536 | [0.659,2.449] | Order *Clostridiales* |
| **New.ReferenceOTU3526** | 1.447 | 1.443 | [0.624,2.253] | Family *Lachnospiraceae* |
| **New.ReferenceOTU1522** | 1.400 | 1.404 | [0.168,2.561] | Family *Ruminococcaceae* |
| **New.ReferenceOTU3977** | 1.353 | 1.350 | [0.498,2.223] | Family *Lachnospiraceae* |
| **New.ReferenceOTU413** | 1.340 | 1.327 | [0.275,2.315] | Family *Ruminococcaceae* |
| **348609** | 1.317 | 1.309 | [0.493,2.123] | Family *Christensenellaceae* |
| **New.ReferenceOTU4280** | 1.269 | 1.247 | [0.044,2.469] | Genus *Ruminococcus* |
| **New.ReferenceOTU3816** | 1.216 | 1.217 | [0.051,2.323] | Family *Ruminococcaceae* |
| **New.ReferenceOTU3320** | 1.203 | 1.209 | [0.376,1.950] | Order *Clostridiales* |
| **New.ReferenceOTU3360** | 1.189 | 1.203 | [0.269,2.049] | Order *Clostridiales* |
| **New.ReferenceOTU1988** | 1.173 | 1.173 | [0.254,2.059] | Order *Clostridiales* |
| **New.ReferenceOTU362** | 1.165 | 1.140 | [0.026,2.341] | Family *Ruminococcaceae* |
| **New.ReferenceOTU1139** | 1.161 | 1.165 | [0.123,2.222] | Family *Lachnospiraceae* |
| **266198** | 1.108 | 1.113 | [0.031,2.120] | Order *RF39* |
| **New.ReferenceOTU591** | 1.102 | 1.107 | [0.174,1.971] | Family *Peptococcaceae* |
| **1108356** | 1.097 | 1.096 | [0.195,2.048] | Order *RF39* |
| **New.ReferenceOTU4631** | 1.096 | 1.106 | [0.123,2.094] | Order *Clostridiales* |
| **New.ReferenceOTU1502** | 1.066 | 1.068 | [0.322,1.717] | Genus *Blautia* |
| **New.ReferenceOTU1728** | 1.055 | 1.067 | [0.098,2.060] | Genus *Coprococcus* |
| **New.ReferenceOTU464** | 1.051 | 1.051 | [0.206,1.950] | Family *Lachnospiraceae* |
| **New.ReferenceOTU3003** | 1.044 | 1.030 | [0.182,1.931] | Family *Ruminococcaceae* |
| **New.ReferenceOTU4382** | 1.029 | 1.035 | [0.159,1.889] | Genus *Faecalibacterium* |
| **359445** | 1.027 | 1.009 | [0.142,1.970] | Family *Ruminococcaceae* |
| **New.ReferenceOTU1835** | 1.027 | 1.024 | [0.305,1.774] | Genus *Coprobacillus* |
| **308760** | 1.022 | 1.022 | [0.137,1.957] | Family *RF39* |
| **New.ReferenceOTU1471** | 1.007 | 1.001 | [0.017,1.940] | Genus *Clostridium* |
| **New.ReferenceOTU1136** | 0.994 | 0.995 | [0.056,1.885] | Family *Ruminococcaceae* |
| **621649** | 0.980 | 0.983 | [0.035,1.884] | Order *Clostridiales* |
| **New.ReferenceOTU3843** | 0.980 | 0966 | [0.059,1.870] | Family *Ruminococcaceae* |
| **New.ReferenceOTU10** | 0.975 | 0.975 | [0.197,1.780] | Family *Lachnospiraceae* |
| **303919** | 0.974 | 0.981 | [0.109,1.801] | Order *Clostridiales* |
| **New.ReferenceOTU2793** | 0.973 | 0.967 | [0.150,1.814] | Family *Ruminococcaceae* |
| **New.ReferenceOTU1738** | 0.966 | 0.956 | [0.244,1.709] | Order *RF39* |
| **New.ReferenceOTU1153** | 0.917 | 0.921 | [0.191,1.693] | Genus *Blautia* |
| **New.ReferenceOTU1425** | 0.906 | 0902 | [0.172,1.660] | Family *Lachnospiraceae* |
| **584263** | 0.847 | 0.847 | [0.020,1.652] | Order *Clostridiales* |
| **New.ReferenceOTU1350** | 0.837 | 0.850 | [0.027,1.622] | Order *Clostridiales* |
| **New.ReferenceOTU1725** | 0.810 | 0.798 | [0.019,1.638] | Order *RF39* |
| **New.ReferenceOTU2473** | 0.795 | 0.770 | [0.009,1.639] | Order *RF39* |
| **New.ReferenceOTU229** | 0.794 | 0.788 | [0.070,1.456] | Family *Lachnospiraceae* |
| **New.ReferenceOTU3779** | 0.779 | 0.782 | [0.032,1.502] | Order *Clostridiales* |
| **New.ReferenceOTU2823** | 0.772 | 0.761 | [0.108,1.449] | Order *RF39* |
| **New.ReferenceOTU4299** | 0.767 | 0.765 | [0.051,1.514] | Order *Clostridiales* |
| **4343981** | 0.762 | 0.772 | [0.026,1.504] | Order *RF39* |
| **279179** | 0.742 | 0.755 | [0.030,1.370] | Order *RF39* |
| **350438** | 0.738 | 0.736 | [0.155,1.285] | Order *Clostridiales* |
| **New.ReferenceOTU918** | 0.734 | 0.740 | [0.112,1.297] | Order *Clostridiales* |
| **New.ReferenceOTU3516** | 0.723 | 0.725 | [0.136,1.348] | Family *Lachnospiraceae* |
| **328083** | 0.701 | 0.699 | [0.041,1.391] | Genus *Clostridium* |
| **216136** | 0.691 | 0.693 | [0.014,1.300] | Order *Clostridiales* |
| **New.ReferenceOTU4525** | 0.667 | 0.673 | [0.030,1.350] | Order *Clostridiales* |
| **New.ReferenceOTU1233** | 0.611 | 0.615 | [0.030,1.196] | Family *Ruminococcaceae* |
| **New.ReferenceOTU1089** | 0.550 | 0.551 | [0.027,1.144] | Genus Ruminococcus |
| **New.ReferenceOTU3335** | 0.452 | 0.449 | [0.085,0.826] | Family *Erysipelotrichaceae* |
| **571111** | -0.707 | -0.695 | [-1.424,-0.025] | Genus Ruminococcus |
| **New.ReferenceOTU4158** | -0.755 | -0.727 | [-1.585,-0.054] | Genus *Rikenella* |
| **537548** | -0.772 | -0.780 | [-1.548,-0.003] | Order *Clostridiales* |
| **331086** | -0.815 | -0.817 | [-1.499,-0.043] | Genus Ruminococcus |
| **364179** | -0.901 | -0.894 | [-1.795,-0.030] | Genus *Bacteroides* |
| **537219** | -0.968 | -0.970 | [-1.902,-0.081] | Order *Clostridiales* |
| **332732** | -1.044 | -1.046 | [-1.869,-0.132] | Genus *Bacteroides* |
| **New.ReferenceOTU2960** | -1.093 | -1.094 | [-1.780,-0.433] | Order *Clostridiales* |
| **New.ReferenceOTU669** | -1.125 | -1.135 | [-2.059,-0.125] | Genus *Methanobrevibacter* |
| **New.ReferenceOTU4624** | -1.145 | -1.147 | [-1.969,-0.325] | Family *Ruminococcaceae* |
| **New.ReferenceOTU3820** | -1.186 | -1.181 | [-2.297,-0.016] | Order *Clostridiales* |
| **New.ReferenceOTU2572** | -1.194 | -1.201 | [-2.156,-0.246] | Genus *Coprobacillus* |
| **860192** | -1.301 | -1.297 | [-2.370,-0.293] | Genus *Coprobacillus* |
| **211066** | -1.314 | -1.310 | [-2.396,-0.189] | Family *Ruminococcaceae* |
| **644244** | -1.423 | -1.421 | [-2.532,-0.394] | Order *Clostridiales* |
| **New.ReferenceOTU4568** | -1.428 | 1.408 | [-2.572,-0.352] | Family *Lachnospiraceae* |
| **522353** | -1.438 | -1.439 | [-2.494,0.406] | Genus *Coprobacillus* |
| **New.ReferenceOTU3611** | -1.481 | -1.476 | [-2.675,-0.233] | Family *Erysipelotrichaceae* |
| **213671** | -1.486 | -1.488 | [-2.559,-0.290] | Family *Rikenellaceae* |
| **New.ReferenceOTU570** | -1.704 | -1.624 | [-3.194,-0.475] | Genus *Phascolarbacterium* |
| **New.ReferenceOTU369** | -1.929 | -1.904 | [-3.224,-0.699] | Genus *Desulfovibrio* |
